# SUPERIOR SVG: no touch saphenous harvesting to improve patency following coronary bypass grafting (a multi-Centre randomized control trial, NCT01047449)

**DOI:** 10.1186/s13019-019-0887-x

**Published:** 2019-05-02

**Authors:** Saswata Deb, Steve K. Singh, Domingos de Souza, Michael W. A. Chu, Richard Whitlock, Steven R. Meyer, Subodh Verma, Anders Jeppsson, Ayman Al-Saleh, Katheryn Brady, Purnima Rao-Melacini, Emilie P. Belley-Cote, Derrick Y. Tam, P. J. Devereaux, Richard J. Novick, Stephen E. Fremes, on behalf of The SUPERIOR SVG Study Investigators

**Affiliations:** 1 Sunnybrook Health Sciences Centre, Institute of Health Policy, Management and Evaluation, University of Toronto, 2075 Bayview Ave., Room H405, Toronto, ON M4N 3M5 Canada; 2 Brigham and Women’s Hospital, Harvard Medical School, Boston, MA USA; 3 Department of Cardiothoracic and Vascular Surgery, Orebro University, Orebro, Sweden; 4 Department of Surgery, Western University, London Health Sciences Centre, London, Canada; 5 Population Health Research Institute, McMaster University and Hamilton Health Sciences, Hamilton, Canada; 6 Mazankowski Alberta Heart Institute, University of Alberta, Edmonton, Canada; 7 St. Michael’s Hospital, Toronto, Canada; 8 Department of Molecular and Clinical Medicine, Institute of Medicine, Sahlgrenska Academy, University of Gothenburg, Gothenburg, Sweden; 9 McMaster University, Hamilton, Canada; 10 University of Calgary and Foothills Medical Centre, Calgary, Canada

**Keywords:** No touch atraumatic saphenous vein graft harvesting, Conventional open saphenous vein graft harvesting, Endoscopic saphenous vein graft harvesting, Multi-centred randomized controlled trial, Coronary artery bypass grafting surgery, Graft patency, Major adverse cardiac and cerebrovascular outcomes

## Abstract

**Background:**

Single centre studies support No Touch (NT) saphenous vein graft (SVG) harvesting technique. The primary objective of the SUPERIOR SVG study was to determine whether NT versus conventional (CON) SVG harvesting was associated with improved SVG patency 1 year after coronary artery bypass grafting surgery (CABG).

**Methods:**

Adults undergoing isolated CABG with at least 1 SVG were eligible. CT angiography was performed 1-year post CABG. Leg adverse events were assessed with a questionnaire. A systematic review was performed for published NT graft patency studies and results aggregated including the SUPERIOR study results.

**Results:**

Two hundred and-fifty patients were randomized across 12-centres (NT 127 versus CON 123 patients). The primary outcome (study SVG occlusion or cardiovascular (CV) death) was not significantly different in NT versus CON (NT: 7/127 (5.5%), CON 13/123 (10.6%), *p* = 0.15). Similarly, the proportion of study SVGs with significant stenosis or total occlusion was not significantly different between groups (NT: 8/102 (7.8%), CON: 16/107 (15.0%), *p* = 0.11). Vein harvest site infection was more common in the NT patients 1 month postoperatively (23.3% vs 9.5%, *p* < 0.01). Including this study’s results, in a meta-analysis, NT was associated with a significant reduction in SVG occlusion, Odds Ratio 0.49, 95% Confidence Interval 0.29–0.82, *p* = 0.007 in 3 randomized and 1 observational study at 1 year postoperatively.

**Conclusions:**

The NT technique was not associated with improved patency of SVGs at 1-year following CABG while early vein harvest infection was increased. The aggregated data is supportive of an important reduction of SVG occlusion at 1 year with NT harvesting.

**Trial registration:**

NCT01047449.

**Electronic supplementary material:**

The online version of this article (10.1186/s13019-019-0887-x) contains supplementary material, which is available to authorized users.

## Introduction

Graft patency is an important determinant of long-term clinical success after coronary artery bypass graft surgery (CABG) [1]. The most common utilized conduit in CABG continues to be the saphenous vein graft (SVG). Contemporary studies continue to show 1-year SVG occlusion rates ranging from 10 to 30% [2–4], and these occlusions are associated with cardiac events.

The atraumatic No Touch technique (NT) of harvesting the SVG with its pedicle intact has been shown to result in favorable biochemical and histological properties of the NT SVG compared to the conventional (CON) SVG technique [5–8]. A single-centre randomized controlled trial (RCT) demonstrated that early [9] (18 months, NT: 95.4%, CON: 88.9%, *p* = 0.03), mid-term [10] (8.5 years, NT 90%, CON: 76%, *p* = 0.01) and late [11] (16 years, NT: 83%, CON: 64%, *p* = 0.03) SVG patency was superior in NT compared to CON SVGs. The evidence in support of this potentially very important treatment effect has been derived from single centre investigations [9–14] to date. According to the 2018 European guidelines on myocardial revascularization [15], the NT technique is now a class IIa recommendation when a SVG is being harvested in an open fashion.

The objectives of the **SU**rgical and **P**harmacological novel int**ER**ventions to **I**mprove **O**verall **R**esults of **S**aphenous **V**ein Graft Patency in Coronary Artery Bypass Grafting surgery trial (SUPERIOR SVG, NCT 01047449) [16] were to determine the effects of the NT compared to the CON technique on graft patency and clinical outcomes 1-year after CABG. Using a factorial design, fish oil supplementation was also compared to placebo. This report will focus on the results of NT SVG harvesting .

## Methods

This was an international, prospective, multi-centre, RCT utilizing a 2 × 2 factorial design involving 12-centres (9 Canada, 2 Sweden, 1 Israel). The complete protocol approved by Health Canada is detailed in Additional file 1. The ethics committees at each participating centre approved the trial, and all participants provided written informed consent.

### Population

Adults > 18 years of age scheduled to undergo isolated elective CABG surgery within 30-days of randomization, left ventricular ejection fraction (LVEF) > 20%, requiring at least one SVG, and estimated glomerular filtration rate (eGFR) > 30 ml/min/1.73m^2^ were included. Additional details are provided in Additional file 1.

### Randomization

Randomization was performed centrally using a web-based computer-generated randomization schedule in a 1:1 ratio, stratified by site using a factorial design (NT vs CON and FO vs P) in variable block sizes to preserve concealment.

### Interventions

Details of the NT or CON interventions are described in Additional file 2. The left anterior descending (LAD) was grafted with an in internal mammary artery and SVGs or other arterial grafts were used for non-LAD target vessels. One study SVG target (NT versus CON based on randomization) was identified by the surgeon; any additional targets and conduits were used at the discretion of the operating surgeon - it was encouraged to use the allocated SVG harvesting method for all SVGs in any particular patient. [Patients participating in the FO factorial received either 2-g/day FO supplement or 2-g of a colour, shape, and taste matched placebo from day 1 of randomization to 1 year after CABG.] All other perioperative medical management was left to the discretion of the attending physician.

### Follow-up

Patients underwent graft assessment using a 64-slice cardiac computed tomography (CCTA) at 1 year after CABG and clinical assessment at 30-days, 3, 6, 9, and 12 months post-operatively.

### Outcomes

The primary outcome was the proportion of study SVGs (NT vs CON) which were completely (100%) occluded on 64-slice CCTA at 1-year following CABG or death due to cardiovascular or unknown causes.

The secondary outcomes were angiographic (the number of study SVGs with a significant (50–99%) stenosis, and a composite of significant stenosis or complete occlusion of the study SVG), and clinical (major adverse cardiac and cerebrovascular events (MACCE: defined as all-cause mortality, non-fatal myocardial infarction (MI) including perioperative MI [17], stroke, and repeat revascularization at 1 year). Leg adverse events (infection, necrosis, dehiscence, drainage, fluid collection) and leg quality of life were assessed with a questionnaire [8].

Detailed definitions of all outcomes can be found in Additional file 2.

CCTAs and MACCE were centrally adjudicated by blinded assessors.

### Statistical analysis

The primary analysis was performed using intention to treat although analysis based on treatment received was also performed for primary, and secondary outcomes. Baseline demographics of the treatment groups were compared in the surgical arm and separately in the pharmacological arm. Normality for continuous variables was tested using the Kolmogorov-Smirnov test – continuous variables were reported as the mean +/− standard deviation or Median (25th–75th percentiles). Categorical variables are reported as the absolute frequency and as a percentage.

*Given the 2 × 2 factorial design, the interaction term between the surgical and pharmacological arm was initially tested for the major primary and secondary outcomes.* The treatment effect for the primary outcome in both arms was estimated using odds ratios (OR) and the corresponding 95% confidence interval (CI) reported.

Categorical variables were compared using the chi-square test or Fisher’s Exact test where appropriate. All continuous variables were compared using the t-test for independent samples if parametric and Wilcoxon rank-sum test if non-parametric. The time to first event for the composite outcome MACCE was tested using the log-rank test. The treatment effect was estimated using the hazard ratio with 95% confidence interval using the Cox proportional hazards model; the proportional hazards assumption was assessed by including a log time-treatment interaction term. A 2-tailed *p*-value of < 0.05 was considered statistically significant. All analyses were performed using SAS, version 9.4 for UNIX (Cary NC, USA). See Additional file 2 for additional description of the statistical analysis, systematic review, meta-analysis and sample size considerations.

## Results

### Patients

**Fig. 1:**
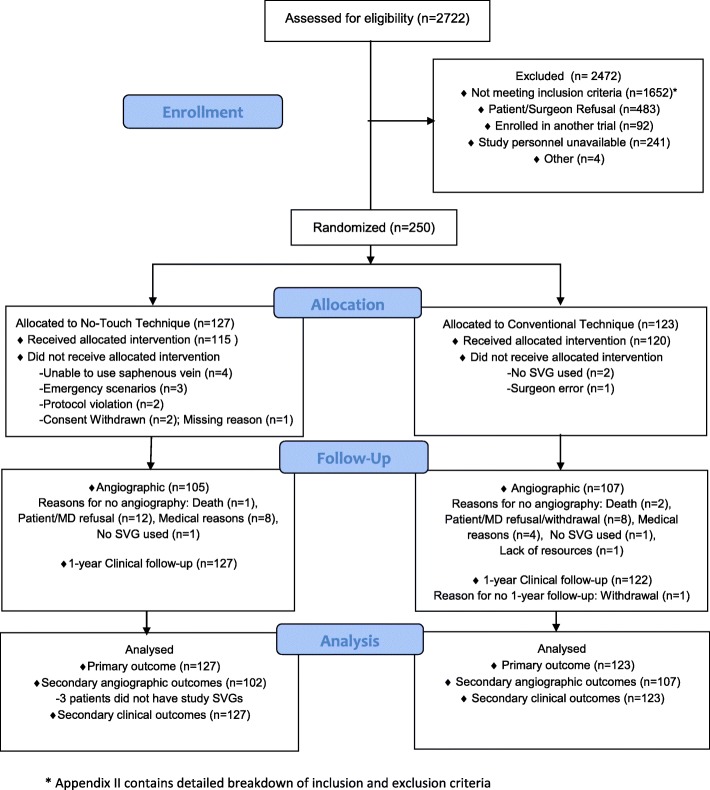
Consort diagram of the surgical arm (No touch versus Conventional technique)

From August 2011 to September 2013, 250 patients of the intended total sample size were enrolled (Fig. 1), as funding for the completion of the trial was not secured. Randomization resulted in 127 and 123 patients in the NT and CON groups, respectively. Fifteen patients (6.0%, NT: 12, CON: 3) did not undergo the surgical technique assigned – individual reasons for non-adherence to the protocol are provided in Fig. 1. Patient details are presented in Additional file 2: Table S1. See Additional file 2: Table S2 for the individual reasons for ineligibility.

### Intra-operative data

Operative characteristics are summarized in Additional file 2: Table S3. The median number of grafts was 3 in each group. The total duration of surgery was longer in the NT group (NT: 5.2 +/− 1.6 h, CON: 4.8 +/− 1.3 h, *p* = 0.02). All other operative characteristics were similar between groups.

### Follow-up

The mean time from CABG to CCTA was 12.7 +/− 2.2 months. In the surgical arm, 212/250 (84.8%, NT: 105, CON: 107) underwent a CCTA (Fig. 1). There was 100% clinical follow-up. Further details pertaining to postoperative management and follow-up can be found in Additional file 2.

### Study outcomes

The interactions between the surgical and pharmacological arms for the predefined primary and secondary outcomes were tested initially due to the factorial design and were non-significant (Table 1). The results for the surgical intervention are reported here.

**Table 1:** Outcomes for the Surgical Arm

	Surgical Arm				
	No Touch (*n* = 127) n (%)	Conventional (*n* = 123) n (%)	Odds Ratio, (95% Confidence Interval)	*p*-value^*^	Interaction^**^p-value
Primary Outcome: Proportion of study SVG with complete occlusion or death from cardiovascular cause at 1 year	7 (5.5)	13 (10.6)	0.49 (0.19–1.28)	0.15	0.35
Proportion of study SVG with complete occlusion^c^	7 (6.9)	11 (10.3)	0.64 (0.24–1.73)	0.38	
Death from cardiovascular cause	0 (0)	2 (1.6)	–	0.24	
Secondary Outcomes^c^					
Proportion of study SVG with significant^b^ stenosis at 1 year	1 (1.0)	5 (4.7)	0.20 (0.02–1.76)	0.12	0.96
Proportion of study SVG with significant^b^ stenosis or complete occlusion	8 (7.8)	16 (15.0)	0.48 (0.20–1.19)	0.11	0.72
MACCE^d^	23 (18.1)	19 (15.4)	1.19 (0.64–2.19)^a^	0.59	0.87
Components of MACCE					
All-cause mortality	1 (0.8)	4 (3.3)	0.24 (0.03–2.14) ^a^	0.20	
Non-fatal MI (MACCE)^d^	19 (15.0)	14 (11.4)	1.33 (0.66–2.67) ^a^	0.43	
Repeat revascularization	2 (1.6)	4 (3.3)	0.47 (0.09–2.57) ^a^	0.38	
Stroke	2 (1.6)	1 (0.8)	1.94 (0.18–21.4) ^a^	0.59	

*Abbreviations*: *MACCE* major adverse cardiac and cerebrovascular events defined by all-cause mortality, non-fatal myocardial infarction, repeat revascularization, or stroke at 1 year

Legend:

^*^Test of significance between No-touch and Conventional, using the logistic regression model for the primary outcome or Fisher’s Exact tests with 0 cells or Cox Proportional hazards model for the MACCE and components of MACCE

^**^Interaction was tested between surgical and pharmacological arm for each of the primary and secondary outcomes

^a^Hazard ratio

^b^Significant stenosis defined by 50–99% stenosis

^c^Note, 102 patients with study SVGs in the No Touch group and 107 patients in the Conventional group underwent 1-year angiogram and therefore contributed to secondary angiographic outcomes

^d^31/33 non-fatal MI (MACCE) events occurred between 0 and 31 days after surgery

#### Primary outcome

The proportion of study SVG which were totally occluded at 1-year or cardiovascular death (primary outcome) was not significantly different in the NT cohort compared to CON (NT: 7/127 (5.5%), CON: 13/123 (10.6%), OR 0.49, 95% CI (0.19–1.28), *p* = 0.15) (Table 1). There were 2/123 (1.6%) cardiovascular deaths in the conventional group and 0 in the NT group. According to the treatment received, the incidence of the primary outcome was 6/116 (5.2%) in the NT patients and 14/127 (11.0%) in the CON group, *p* = 0.10.

#### Secondary outcomes

**Fig. 2:**
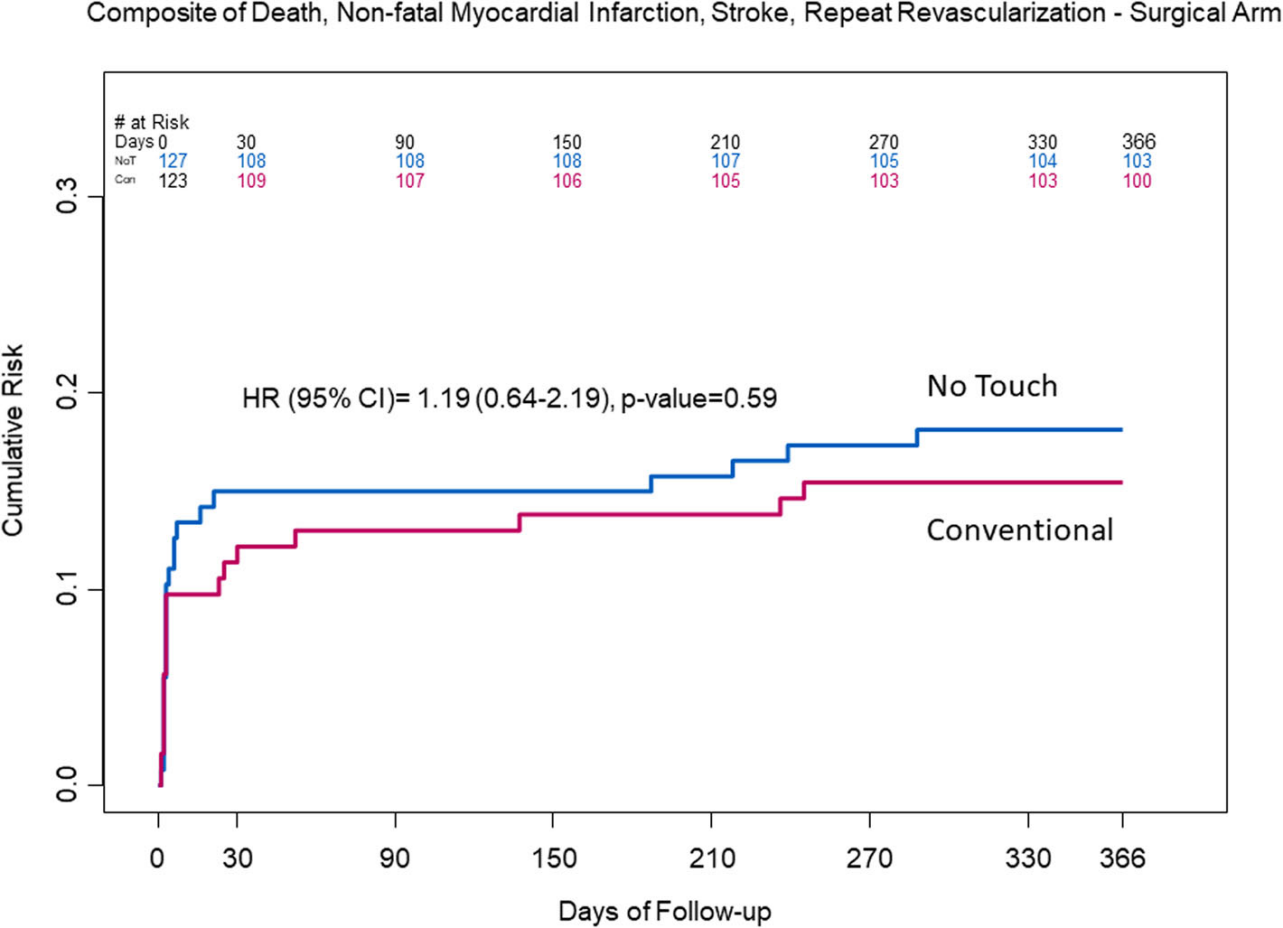
Kaplan Meier plot of the secondary outcome, MACCE (major adverse cardiac and cerebrovascular events (death, non-fatal myocardial infarction, stroke, repeat revascularization)), between the No Touch and Conventional groups

The proportion of study SVGs with significant stenosis (NT: 1/102 (1.0%), CON 5/107 (4.7%), *p* = 0.12) and the composite of significant stenosis or complete occlusion (NT: 8/102 (7.8%), CON: 16/107 (15.0%), *p* = 0.11 were not significantly different in the NT compared to CON (Table 1); there was a higher, but not significant proportion in the CON group according to treatment received (NT: 7/98 (7.1%), CON: 17/111 (15.3%), *p* = 0.06). The proportion of MACCE was similar between NT (23/127 (18.1%) and CON (19/123 (15.4%) (*p* = 0.59); most of the events were driven by non-fatal perioperative MI (NT 19/23, CON 14/19) (Fig. 2).

#### Additional graft analysis

Additional file 2: Table S4 provides additional descriptive information regarding the size and quality of the grafts and targets in the NT and CON groups, which were well balanced between groups. Table 2 describes the SVG complete occlusion, and significant stenosis or complete occlusion results for all SVGs, study and non-study, according to the treatment received analysis. Endoscopic veins were not included in the analysis. While the number of total SVGs in the NT arm (267) and CON groups (269) were almost identical (Additional file 2: Table S3), there were 282 conventional SVGs altogether and only 214 NT SVGs, indicating that conventional (or endoscopic) SVGs were used for many of the non-study SVGs in the NT group. According to generalized estimating equations models, the proportion of graft occlusion was reduced but not significantly different in the NT SVGs, while stenosis or graft occlusion was decreased significantly in the NT SVGs (*p* = 0.02) compared to the CON SVGs.

**Table 2:** Graft Status for all SVGs – Treatment Received Analysis

Outcome	#Grafts in No Touch *N* = 191	#Grafts in Conventional *N* = 243	No Touch vs Conventional Odds Ratio (95% Confidence Interval)	*p*-value	Interaction *p*-value
Total Occlusion	18 (9.4%)	36 (14.8%)	0.60 (0.32–1.12)	0.107	0.998
50–99% Stenosis or Total Occlusion	19 (9.9%)	46 (18.9%)	0.49 (0.27–0.88)	0.018	0.933

Legend: Chi-square p values are from the generalized estimating equations model results. OR = odds ratio. CI = confidence interval. The interaction *p* value refers to the interaction between the numbers of grafts in each patient and treatment received. Endoscopic veins are excluded

#### Subgroup analysis

**Fig. 3:**
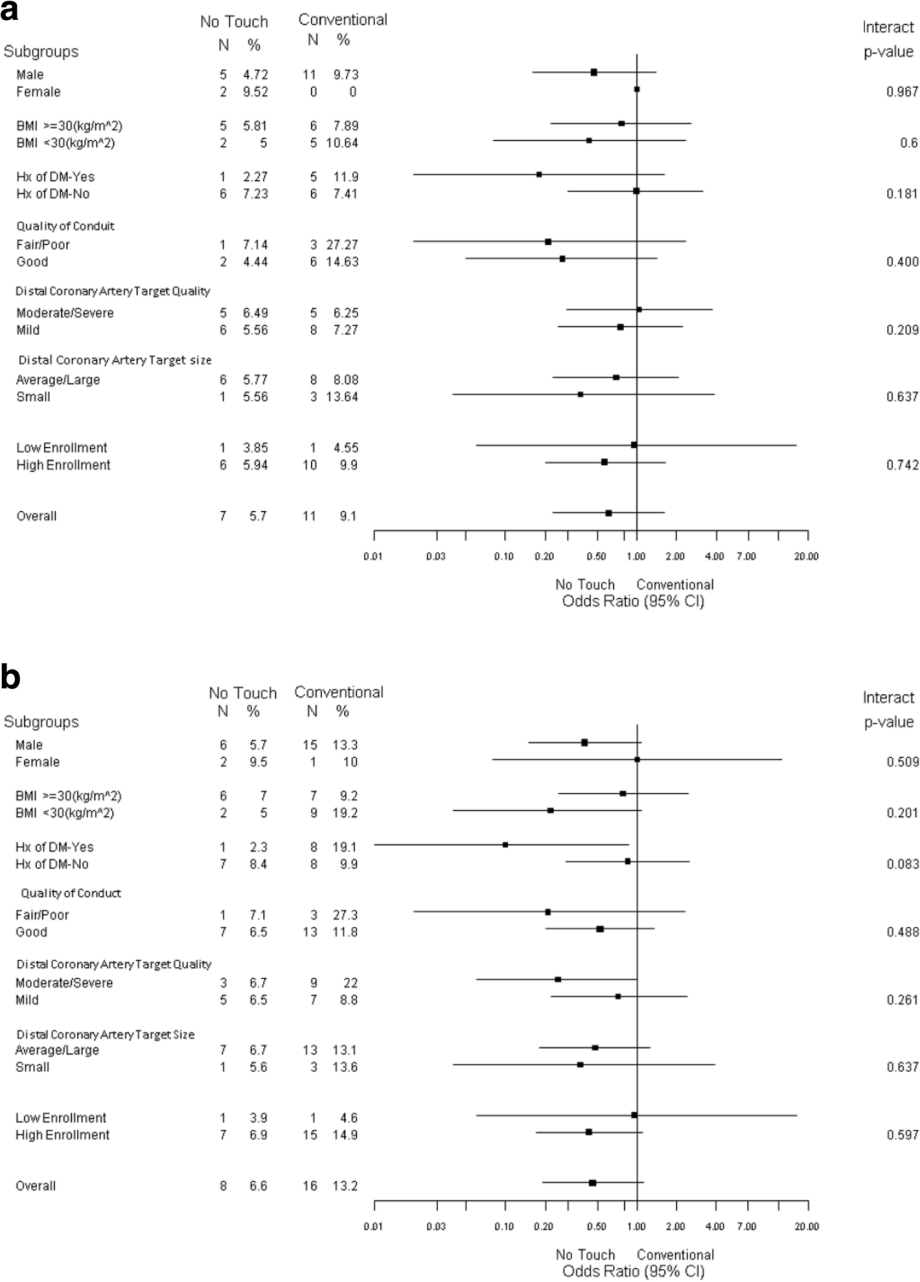
**a** Forest plot for early vein graft occlusion at median 1 year reported follow-up between No Touch technique and Conventional SVG harvesting technique. The graft occlusion results from the current study refer to the study saphenous veins according to the allocated treatment. **b** Forest plot for early vein graft occlusion or significant stenosis (> 50% narrowing of lumen) at median 1 year reported follow-up between No Touch technique and Conventional SVG harvesting technique. The graft occlusion results from the current study refer to the study saphenous veins according to the allocated treatment

Subgroup analyses were performed for complete occlusion (Fig. 3a) and significant and/or complete occlusion (Fig. 3b). All interaction *p* values were non significant between the subgroups and the treatment group. The OR for complete occlusion among diabetics undergoing a NT harvesting technique was 0.18, 95% CI (0.02–1.62); for significant and/or complete occlusion, the OR was 0.10, 95% CI (0.01–0.87).

#### Adverse surgical events

There were no significant differences between other adverse surgical events between the NT and CON technique (Additional file 2: Table S6).

#### Leg assessment

The proportion of leg infections was higher in the NT group at 30 days (NT 27/116 (23.3%), CON 11/116 (9.5%), *p* < 0.01), as was the severity (*p* = 0.004, Table 3). By 1 year, the prevalence and severity was similar between the 2 groups (Table 3). The cumulative incidence of wound infection at 1 year was 31 (25.4%) and 14 (11.8%) in the NT and CON groups respectively. Other wound assessment results are presented in Additional file 2: Table S7. Adverse leg outcomes were all greater in the NT legs. The Adverse SVG Harvesting Score, Leg Quality of Life Score and Total Leg Scores were all significantly greater in the NT legs at 1 and 3 months but similar and low at 1 year postoperatively. According to the mixed level model analysis (Table 4), the Total Leg Scores are higher in the No Touch group as compared to the Conventional group at all of the times (interaction *p* value 0.09). This was similar for adjusted total leg scores (interaction between treatment and time *p* = 0.06) (adjusted by gender, history of diabetes, history of PAD, BMI groups, low vs high enrollment centers).

**Table 3:** Leg Infection

	NT	CON	*P*-value
Incidence of Infection, n (%)			
30-days: *N* = 232	27 (23.3)	11 (9.5)	
3-months: *N* = 206	10 (9.4)	3 (3.0)	
1-year: *N* = 222	1 (0.9)	1 (0.9)	
Cumulative Incidence at 1 year: *N* = 241	31 (25.4	14 (11.8)	0.007
Severity of Infection (Median (Q1-Q3))			
30-days *N* = 232	0 (0–0)	0 (0–0)	
3-months: *N* = 206	0 (0–0)	0 (0–0)	
1-year: *N* = 222	0 (0–0)	0 (0–0)	

Legend: Incidence and severity of infection. For severity of infection, the score ranges from 0 to 10 with a higher score being a more severe state (see Additional file 2 for more details)

**Table 4:** Mixed Level Model Results for the Total Leg Score

Time	No Touch LS Means (95% CI)	Conventional LS Means (95% CI)	Adjusted difference (No Touch – Conventional)*	*P*-value for treatment difference*	*P*-value for interaction (treatment by time)
30 Days	6.44 (5.64–7.25)	3.86 (3.06–4.67)	2.58 (0.920–4.24)	0.0002	
3 Months	4.49 (3.67–5.32)	2.20 (1.35–3.04)	2.30 (0.58–4.02)	0.0021	
1 Year	2.77 (1.96–3.58)	1.65 (0.82–2.48)	1.12(−0.57–2.81)	0.407	
					0.094

Legend: The Total Leg Scores were higher in the NT patients compared to the Control patients, according to linear mixed model analysis. The reported results are the least square means. The range for the Total Leg Score is 0–57 (see Additional file 2 for more details)

*Confidence intervals and *p*-values adjusted for multiple comparisons using Tukey’s method

### Systematic review and meta-analysis

**Fig. 4:**
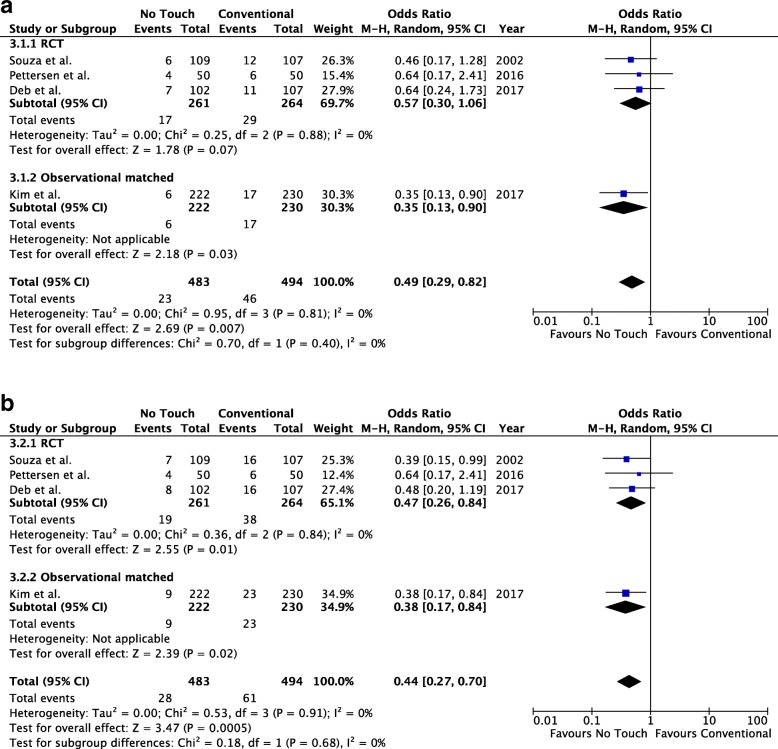
**a** Meta-analysis of no touch versus conventional saphenous vein graft harvesting technique for the outcome of any graft occlusion. **b** Meta-analysis of no touch versus conventional saphenous vein graft harvesting technique for the outcome any graft occlusion or significant stenosis

The literature yielded 295 citations, of which 3 (2 randomized and 1 observational) compared a No-Touch technique to conventional saphenous vein harvesting on our outcomes of interest [9, 12, 14]. Additional file 2: Table S8 summarizes the characteristics of the different studies. There were 483 and 494 NT vs. CON SVGs in total including the SUPERIOR study results for the study grafts according to the intention to treat. The use of NT was protective for the outcomes of any graft occlusion (OR 0.49, 95% CI 0.29–0.82, *p* = 0.007, Fig. 4a) and any graft occlusion or significant stenosis (OR 0.44, 0.27–0.70, *p* = 0.0005, Fig. 4b), without heterogeneity, I^2^ = 0 for both endpoints.

## Discussion

Arterial grafting has been demonstrated to be associated with better long term survival in observational studies [18], and society guidelines recommend increased utilization of arterial grafting [15, 19]. To date, the findings in support of arterial grafts has been mixed in RCTs [20, 21]. The saphenous vein remains the most commonly used bypass graft for coronary bypass surgery. Therefore, strategies to enhance SVG patency has the potential to improve long-term CABG.

No Touch SVG harvesting has been shown to have favorable biochemical and histological findings in vein specimens [5–8] – furthermore, the fatty pedicle may provide some external support, and also reduces the risk of kinking of the SVG in the pericardium [7, 22]. Using an artificial external support device such as a Venous External Stent (VEST) has also shown to reduce diffuse intimal hyperplasia 1 year after CABG [23].

Single centre studies reported results supporting the NT hypothesis [11]. This is the first multi-centre RCT to assess the No Touch SVG compared to the conventional SVG harvesting technique for 1 year outcomes. Collaborating centres received training by two of the investigators familiar with the technique (DDS, SEF). Study SVG occlusion, and study SVG stenosis or occlusion, were lower, but not significantly different, in the NT SVGs in the current study. When all SVGs were considered, the on-treatment analysis of graft stenosis or occlusion was significantly reduced in the NT SVGs suggesting biological superiority. The aggregated data does show a clinically important reduction in graft occlusion (Fig. 4a) and graft stenosis or occlusion (Fig. 4b). The point estimates from this current study are also consistent with the earlier single centre studies without heterogeneity. Furthermore and importantly, graft occlusion of the NT veins to non-LAD targets and internal mammary grafts to the LAD at 16 years in the original Swedish study was not statistically different [11].

Leg infections were significantly higher in the NT legs compared to CON at 30-days. The inclusion criteria for this study were less restrictive than the original study from Sweden [9]. The other adverse events, as well as the adverse event scores, leg quality of life scores and total leg scores were all increased in the NT legs within the first 3 months of surgery, but similar by 1 year. The avoidance of NT vein harvesting may be appropriate particularly for patients with multiple risk factors for surgical site infections. No-touch veins were harvested with an open technique – the use of skin bridges and/or drains as described by Kim and colleagues [14] may lead to a reduction in the incidence of leg wound infections. Insulin dependent diabetes was an exclusion criteria in the Norwegian study, and the body mass index was in the normal range; the incidence of wound infection was low in the NT patients and comparable with the CON [13]. We reported leg wound complications according to the intention to treat analysis as less biased – the treatment received analysis may be more relevant for leg complications.

One of the strengths of this trial is that it was a multi-centre, international RCT. All outcomes were analyzed in a blinded and standardized manner by a central adjudication committee and all angiograms were read centrally by imaging experts blinded to all interventions. We also achieved 100% clinical follow-up and 84.8% underwent CCTA. A limitation of graft patency studies is that the primary outcome is determined only in those who undergo follow-up angiography - all the patients in this study contributed to the primary outcome as cardiovascular death was included in the composite endpoint.

### Limitations

The most significant limitation of this study is that recruitment was substantially less than the planned enrollment. One of the reasons is that the sample size was originally determined assuming a much more conservative treatment effect than what was observed in the Swedish study [9]. Testing for a more modest although still clinically meaningful treatment effect minimizes the risk of a type 1 error, but at the expense of a larger sample size. Post hoc, a much smaller total sample size would have sufficed to test for the surgical hypothesis primary endpoint. Finally, while most of the NT SVG harvesting were performed by staff surgeons or assistant physicians, there may have been a learning curve to this new technique that was not objectively captured in this study.

## Conclusions

No Touch saphenous vein harvesting technique was not associated with superior graft patency or clinical outcomes after CABG. Due to the sample size, the trial cannot exclude a meaningful improvement of graft patency with the NT technique; however the findings are consistent with other trials and the meta-analysis of the aggregated data suggests a reduction in graft stenosis or graft occlusion. However, NT SVG harvesting was associated with an increase in surgical site infection and other leg adverse events. Two ongoing multicentre trials – one in China (NCT03126409) and another in Sweden (NCT03501303) will help to further clarify the role of the No Touch saphenous vein in coronary surgery. Furthermore, studies with longer term follow-up (5 years or more) are also required.

## Additional files

Additional file 1: Contains the Trial Protocol. (PDF 488 kb)

Additional file 2: Contains additional data regarding trial methodology, definitions, and supplementary results. (DOCX 57 kb)

## Abbreviations

CABG: Coronary artery bypass grafting
CCTA: Cardiac computed tomography angiography
CI: Confidence interval
CON: Conventional saphenous vein graft harvesting
CV: Cardiovascular
eGFR: estimated glomerular filtration rate
F: Fish oils
LVEF: Left ventricular ejection fraction
MACCE: Major adverse cardiac and cerebrovascular events
MI: Myocardial infarction
NT: No touch saphenous vein graft harvesting
P: Placebo
RCT: Randomized control trial
SVG: Saphenous vein graft

## References

[1] Buxton BF, Hayward PAR, Newcomb AE, Moten S, Seevanayagam S, Gordon I (2009). Choice of conduits for coronary artery bypass grafting: craft or science?. Eur J Cardiothorac Surg.

[2] Domanski MJ, Borkowf CB, Campeau L (2000). Prognostic factors for atherosclerosis progression in saphenous vein grafts: the postcoronary artery bypass graft (post-CABG) trial. Post-CABG trial Investigators. J Am Coll Cardiol.

[3] Prevent IV Investigators (2005). Efficacy and safety of edifoligide, an e2f transcription factor decoy, for prevention of vein graft failure following coronary artery bypass graft surgery: prevent iv: a randomized controlled trial. JAMA..

[4] Desai ND, Cohen EA, Naylor CD, Fremes SE (2004). A randomized comparison of radial-artery and saphenous-vein coronary bypass grafts. N Engl J Med.

[5] Johansson BL, Souza DS, Bodin L (2010). Slower progression of atherosclerosis in vein grafts harvested with ‘no touch’ technique compared with conventional harvesting technique in coronary artery bypass grafting: an angiographic and intravascular ultrasound study. Eur J Cardiothorac Surg.

[6] Dreifaldt M, Souza DS, Loesch A (2011). The “no-touch” harvesting technique for vein grafts in coronary artery bypass surgery preserves an intact vasa vasorum. J Thorac Cardiovasc Surg.

[7] Dashwood MR, Savage K, Tsui JC (2009). Retaining perivascular tissue of human saphenous vein grafts protects against surgical and distension-induced damage and preserves endothelial nitric oxide synthase and nitric oxide synthase activity. J Thorac Cardiovasc Surg.

[8] Verma S, Lovren F, Pan Y (2014). Pedicled no-touch saphenous vein graft harvest limits vascular smooth muscle cell activation: the PATENT saphenous vein graft study. Eur J Cardiothorac Surg.

[9] Souza DS, Dashwood MR, Tsui JC (2002). Improved patency in vein grafts harvested with surrounding tissue: results of a randomized study using three harvesting techniques. Ann Thorac Surg.

[10] Souza DS, Johansson B, Bojö L, Karlsson R, Geijer H, Filbey D (2006). Harvesting the saphenous vein with surrounding tissue for CABG provides long-term graft patency comparable to the left internal thoracic artery: results of a randomized longitudinal trial. J Thorac Cardiovasc Surg.

[11] Samano N, Geijer H, Liden M, Fremes S, Bodin L, Souza D (2015). The no-touch saphenous vein for coronary artery bypass grafting maintains a patency, after 16 years, comparable to the left internal thoracic artery: a randomized trial. J Thorac Cardiovasc Surg.

[12] Pettersen O, Wiseth R, Hegbom K, Nordhaug DO (2016). Pedicled vein grafts in coronary surgery exhibit reduced intimal hyperplasia at 6 months. J Am Coll Cardiol.

[13] Pettersen O, Haram PM, Winnerkvist A (2017). Pedicled Vein Grafts in Coronary Operation: Perioperative Data From a Randomized Trial. Ann Thorac Surg.

[14] Kim YH, Oh HC, Choi JW, Hwang HY, Kim KB (2017). No-touch saphenous vein harvesting may improve further the patency of saphenous vein composite grafts: early outcomes and 1-year angiographic results. Ann Thorac Surg.

[15] Neumann FJ, Sousa-Uva M, Ahlsson A (2018). 2018 ESC/EACTS Guidelines on myocardial revascularization. Eur Heart J.

[16] SUPERIOR SVG. Improving the Results of Heart Bypass Surgery Using New Approaches to Surgery and Medication (SUPERIORSVG): ClinicalTrials.gov: National Institutes of Health; 2014.

[17] Thygesen K, Alpert JS, Jaffe AS (2012). Third universal definition of myocardial infarction. Circulation..

[18] Gaudino M, Di Franco A, Rahouma M, et al. Unmeasured confounders in observational studies comparing bilateral versus single internal thoracic artery for coronary artery bypass grafting: a meta-analysis. J Am Heart Assoc. 2018;7(1). 10.1161/JAHA.117.008010 PMC5778975 29306899

[19] Aldea GS, Bakaeen FG, Pal J (2016). The Society of Thoracic Surgeons clinical practice guidelines on arterial conduits for coronary artery bypass grafting. Ann Thorac Surg.

[20] Taggart DP, Altman DG, Gray AM (2016). Randomized trial of bilateral versus single internal-thoracic-artery grafts. N Engl J Med.

[21] Gaudino M, Benedetto U, Fremes S, et al. Radial-artery or saphenous-vein grafts in coronary-artery bypass surgery. N Engl J Med. 2018; [Epub ahead of print]. 10.1056/NEJMoa1716026 29708851

[22] Samano N, Dashwood M, Souza D (2018). No-touch vein grafts and the destiny of venous revascularization in coronary artery bypass grafting—a 25 th anniversary perspective. Ann Cardiothoracic Surg.

[23] Taggart DP, Ben Gal Y, Lees B (2015). A randomized trial of external stenting for saphenous vein grafts in coronary artery bypass grafting. Ann Thorac Surg.

