# Supplementary material for: SUPERIOR SVG: no touch saphenous harvesting to improve patency following coronary bypass grafting (a multi-Centre randomized control trial, NCT01047449)
Source: J Cardiothorac Surg. 2019 May 2;14:85. doi: 10.1186/s13019-019-0887-x (PMC6498551; doi:10.1186/s13019-019-0887-x)
Supplement: Supplementary file 1 — Contains the Trial Protocol. (PDF 488 kb) [file 13019_2019_887_MOESM1_ESM.pdf]

Appendix 1: Trial Protocol

FINAL PROTOCOL

SUPERIOR SVG Study

SUrgical and ***P***harmacological novel int***ER***ventions to ***I***mprove ***O***verall ***R***esults of ***S***aphenous ***V***ein ***G***raft Patency in Coronary Artery Bypass Grafting surgery: An International Multi-center Randomized Controlled Clinical Trial

Original Date: December 22, 2009
Formatted Date: March 26, 2010
Amended Date: June 9, 2010 (Amendment 1.0)
Amended Date: June 13, 2011 (Amendment 2.0)

Study Coordinating Group: Population Health Research Institute
DBCVSRI, 237 Barton Street East
Hamilton, Ontario, L8L 2X2
Canada

Sponsor: CANNeCTIN
Heart & Stroke Foundation of Canada

This protocol is the confidential intellectual property of the Study Steering Committee. The use of any unpublished material presented in this document must be restricted to the recipient for the agreed purpose and must not be disclosed to unauthorized persons without the written consent of the SUPERIOR SVG Steering Committee.

STUDY SYNOPSIS

Title	SUPERIOR SVG Trial
Investigator	Dr. Stephen Fremes and Dr. Richard Novick
Study Objective(s)	We will perform an international multi-center factorial design randomized controlled clinical trial, to determine whether a novel atraumatic pedicled (no touch) technique of SVG harvesting (**Surgical Arm**) and/or the use of oral fish oil supplements (**Pharmacological Arm**) improve angiographic patency and clinical outcomes at 1 year, compared to conventional SVG harvesting and placebo, respectively. The objective of the pilot phase of the study is to determine the number of eligible patients of the total isolated CABG patients, the consent rate, adherence to the protocol including rate of follow-up cardiac CT angiography, medication discontinuation and the overall incidence of the primary outcome.
Study Design	This is an international, multi-centre, prospective, controlled RCT using a 2x2 factorial design; involving a surgical intervention and a pharmacological intervention.
Study Population<br>Main selection criteria:	**Inclusion Criteria:**<br>i. Age >18 years<br>ii. Able to provide informed consent<br>iii. Isolated CABG, non-emergent, on- or off-pump (cardiopulmonary bypass)<br>iv. Primary or re-do CABG (if re-do, all previous grafts must be occluded)<br>v. Left ventricular ejection fraction >20%<br>vi. Require at least one SVG as part of revascularization strategy<br>vii. Creatinine clearance of at least 30 mL/min or higher<br><br>**Exclusion Criteria:**<br>i. Unable to use greater SV due to previous vein stripping or poor quality on mandatory preoperative Duplex study and vein mapping<br>ii. Contraindication to receiving cardiac CT angiography (allergy to contrast dye, renal failure with a creatinine >180 µmol/L, uncontrolled atrial fibrillation precluding proper gating of study)<br>iii. Pregnant women, or women of child-bearing age<br>iv. Allergy to fish oil/fish products, and non-medicinal ingredients of the study product (corn oil, soybean oil, gelatine, glycerol, or carob colouring)<br>v. Already taking fish oil supplements regularly (daily use in past 30 days)<br>vi. Congenital or acquired coagulation disorders<br>vii. Patients considered to be of excessive risk of wound infection according to the **<u>clinical judgment of the site surgical investigator</u>**. N.B. Patients that are excluded for anticipated excessive risk of wound infection need to have the reasons for exclusion well documented in the screening log. We expect that the excluded patients will have advanced comorbidities

	or multiple risk factors for wound infection (such as peripheral vascular disease, peripheral edema, diabetes mellitus, obesity, advanced age, malnutrition, steroid dependence, concurrent infection, concurrent malignancy, or frailty).
Total expected number of subjects:	1,550 patients (50 for the pilot study)
Expected number of centres:	This study will be conducted at approximately 10 Canadian and 40 International centers. The pilot phase will involve 5 Canadian and 5 International centres.
Study Drug(s) or Intervention(s)	Each patient will be randomized to a SVG harvested in the conventional fashion (open or endoscopic) or using the no touch technique (single-blinded). The pharmacological arm of the factorial design will also have patients randomized to fish-oil or placebo (double-blinded) which will be started preoperatively and continued for 1 year postoperatively.
Evaluation Criteria	The objective of the pilot phase of the study is to determine the number of eligible patients of the total isolated CABG patients, the consent rate, adherence to the protocol including rate of follow-up cardiac CT angiography, medication discontinuation and the overall incidence of the primary outcome. Achieving a recruitment rate of 1-2 patients/centre/month will determine if the study will proceed to the full phase III trial.<br><br><u>Primary Outcome</u><br>**Surgical Arm**: Proportion of study SVGs ("no touch" versus conventionally harvested) which are totally (100%) occluded on cardiac CT angiography at 1-year post-CABG and death due to cardiovascular or unknown causes.<br>**Pharmacological Arm**: Proportion of patients with ≥1 graft (saphenous or arterial) totally (100%) occluded on cardiac CT angiography at 1-year post-CABG and death due to cardiovascular or unknown causes, comparing the fish-oil to placebo groups.<br><br><u>Secondary Outcome</u><br>**Surgical Arm** are to determine whether:<br>i. The "no touch" technique of SVG harvesting results in a lower ***proportion of study SVGs*** that have a significant stenosis (50-99%) on 1-year post-CABG angiography compared to conventional SVG harvesting techniques.<br>ii. The incidence and severity of adverse SVG harvesting events at 1-year post-CABG (infection, haematoma, swelling, neuropathy, quality of life measures) are similar between the "no touch" and conventional groups of patients.<br>iii. The incidence of the composite of non-fatal MI (using new definition), all-cause mortality, and repeat revascularization (redo CABG or PCI) perioperatively and at 1 year is lower in the "no touch" versus conventional group of patients.<br><br>**Pharmacological Arm** are to determine whether:<br>i. Fish oil supplementation results in a lower ***proportion of***

	patients with ≥1 graft with a significant (50-99%) stenosis at 1-year post-CABG angiography, compared to those who received placebo.<br>ii. The incidence of the composite of non-fatal MI (using new definition), all-cause mortality, and repeat revascularization (redo CABG or PCI) perioperatively and at 1 year is lower in the fish oil versus placebo group of patients.<br><br><u>Tertiary Outcome</u><br>**Surgical Arm**: The two groups will be compared with respect to the incidence of the composite endpoint of perioperative and 1-year non-fatal MI (using old definition), all-cause mortality, repeat revascularization (redo CABG or PCI) (major adverse cardiac events, MACE) and stroke.<br><br>**Pharmacological Arm**: The two groups will be compared with respect to the incidence of the composite endpoint of perioperative and 1-year non-fatal and fatal MI (using old definition), death, repeat revascularization (redo CABG or PCI) (major adverse cardiac events, MACE) and stroke.
Statistical Considerations	Intention to treat analysis<br>Comparison of the proportion of patients developing the primary outcome at 1-year using Pearson chi-square test.<br>Overall type 1 error rate at 5%
Duration of Study Period<br>(per subject)	The study will take 5 years to complete. The pilot phase will take 2 years to complete. The duration of the study period per subject will be 1 year post-CABG.
Anticipated Study Dates:<br>(start/end)	January 2010 is the anticipated start date and December 2014 is the anticipated end date.

LIST OF ABBREVIATIONS

**Abbreviation**	**Definition**
AE	Adverse event
AF	Atrial fibrillation
CABG	Coronary artery bypass grafting
CCS	Canadian Cardiovascular Society
CCTA	Cardiac computer tomography angiography
CK-MB	Creatine kinase myocardial b fraction
CPB	Cardiopulmonary bypass
CRF	Case report form
CV	Cardiovascular
DHA	Docosahexaenoic acid
DSMB	Data safety monitoring board
ECG	Electrocardiogram
eGFR	Estimated Glomerular Filtration Rate
EHA	Eicosapentaenoic acid
GCP	Good Clinical Practice
ICU	Intensive care unit
IRB	Institutional Review Board
IVRS	Interactive voice activated randomization system
LA	Left atrium
LITA	Left internal thoracic artery
LV	Left ventricle
LVEF	Left ventricular ejection fraction
MACE	Major adverse cardiac event
MI	Myocardial infarction
NSAIDS	Non-steroidal anti-inflammatory drugs
OAC	Oral anticoagulant
PCI	Percutaneous coronary intervention
PHRI	Population Health Research Institute
RA	Radial artery
RBC	Red blood cells
RCT	Randomized controlled trial
REB	Research Ethics Board
RR	Relative risk
SAE	Serious adverse event
SV	Saphenous vein
SVG	Saphenous vein graft

CONTENTS

List of Abbreviations 7
1 Introduction and Rationale 10
A. INTRODUCTION 10
2 Study Objectives 13
3 Study Design 14
3.1 Type of study 14
3.2 Expected number of subjects 15
3.3 Method of treatment allocation 15
3.4 Duration of the study period for each subject 15
4 Study Population 15
4.1 Inclusion Criteria 15
4.2 Exclusion Criteria 16
5 Study Procedures 16
5.1 Treatments 16
5.2 Schedule of visits and observations 17
5.3 Selection procedures (entry procedures) 19
5.4 Encouraging compliance 19
5.5 Study measurements 20
Study Outcomes 20
Definitions of Study Outcomes 22
6 Supplement Supplies 24
6.1 Study Supplements 24
6.2 Packaging and labeling 24
6.3 Storage conditions 24
6.4 Responsibilities 24
7 Subject Safety 25
7.1 Monitoring of adverse events 25
7.2 Safety instructions specific to the trial 25
Emergency Unblinding 26
7.3 Premature Withdrawal/Discontinuation Criteria 26
8 Statistical Considerations 26
8.1 Study outcomes 26
8.2 Analysis population 26
8.3 Statistical Methods 27
8.4 DSMB and interim analysis 27
8.5 Sample size calculation 28
9 Study Organization 29
9.1 Coordinating Centre 29
9.2 National Coordinators 29
9.3 Event Adjudication Committee 30
10 Ethical and Regulatory Standards 30
10.1 Regulatory and ethical considerations 30
10.2 Laws and regulations 30
10.3 Informed consent 30
10.4 Ethics Review Committee 31
11 Study Monitoring 31
11.1 Responsibilities of the investigator(s) 31

11.2 Data collection on case report forms (CRFs) 31
12 Administrative Rules 32
12.1 Curriculum vitae 32
12.2 Record retention in investigating centre(s) 32
13 Ownership of Data and Use of the Study Results 32
14 Publications 32
15 Protocol Amendments 33
16 Bibliographic References 34

1 INTRODUCTION AND RATIONALE

A. INTRODUCTION

The SUPERIOR SVG trial is a prospective, international, multi-centre randomized controlled clinical trial (RCT). It utilizes a factorial design to elucidate the benefits of two interventions, one surgical and the other pharmacological. Both impact an important, contemporary clinical problem – poor saphenous vein graft (SVG) patency after coronary artery bypass grafting surgery (CABG).

I. The problem to be addressed

According to serial angiographic observational studies and pivotal trials on the benefit of antiplatelet medications on SVG patency, 1-year SVG occlusion rates are estimated to be 10-15% (1-4). Contemporary studies demonstrate SVG failure remains a significant issue of even greater importance than in previous decades. The PREVENT IV multi-centre trial (JAMA, 2005) enrolled 3,014 CABG patients - one of the largest CABG trials ever, *the* largest CABG trial with angiographic follow-up (5). The authors found at 1 year: 45% of ***all patients*** had >1 SVG occluded; 25% of ***all SVGs*** were occluded. Recent single centre trial data from Sunnybrook evaluating the role of intraoperative Graft Imaging to Improve Patency (GRIIP) also demonstrated that 30% of all patients have ≥1 SVG occluded by ~1 year (6). In contrast, the multi-centre Radial Artery Patency Trial (RAPS), compared the patency of the RA to SVG in patients who served as their own controls (7). The results showed 8% of radial artery bypass conduits and 13% of SVG conduits, were occluded at 1-year angiography – however these occlusions were in study vein grafts, implying a much higher rate of occlusion if the non-study grafts had been included. Perhaps more importantly, RAPS patients were largely recruited from the 1990's whereas PREVENT IV and GRIIP patients were enrolled since 2000. Furthermore, the importance of graft patency is highlighted from the results of PREVENT IV; patients with at least one SVG occluded had two times the incidence of perioperative myocardial infarction (MI), and thirteen times the composite endpoint of death, late MI or repeat revascularization at 12-18 months, compared to patients who had completely patent grafts (5).

II. The need for this trial

Early and late success of CABG is the result of sustained patency of the bypass grafts. Graft failure due to intimal hyperplasia and the progression of atherosclerosis is a complex, multifactorial inflammation process. It is initiated largely in areas of endothelial injury, secondary to biomechanical disturbances or trauma, or systemic factors such as lipoprotein disturbances and endothelial dysfunction. Accumulating data indicate that structural and/or functional alterations in endothelial integrity play a primordial role in the development of atherosclerosis via promoting aberrant interactions between modified lipoproteins, macrophages and other cellular elements of the vascular wall, inciting early plaque formation. As such, ***strategies, either pharmacological or mechanical in nature, which reduce and/or restore endothelial homeostasis remain an important cornerstone to limit atherosclerosis and vascular graft failure.***

While definitely more invasive, historically CABG has been more effective and durable than percutaneous coronary intervention (PCI) for the treatment of left main and multivessel coronary artery disease (8-10). However, the results (7-17), indications (16-17) and volume of PCI have expanded significantly, such that patients referred for CABG have more challenging coronary

anatomy, with diffuse, severe, small vessel (<2 mm), calcific disease. It is this recent change in the target vessels in contemporary CABG patients, which most likely explains the apparent decrease in 1-year SVG patency over the last decade (18).

This study aims to favourably impact SVG patency by integrating surgical and pharmacological approaches which address both the mechanical and biochemical pathways contributing to accelerated atherosclerosis, subsequent graft failure and poor clinical outcomes; we have designed a factorial 2x2 randomized controlled clinical trial to efficiently test both interventions.

B. BACKGROUND AND RATIONALE

Early landmark clinical trials have demonstrated the benefits of early administration of antiplatelet medications (primarily aspirin) to enhance saphenous vein graft patency, at a minimum for the first year post-CABG (1,2). These studies established that the 1-year expected saphenous vein graft occlusion rate is approximately 10-15% (1-3). More recent data has shown that long-term adherence to statin medications, with anti-lipid and anti-inflammatory properties, also significantly improves early, mid-term and late graft patency (19,20). Intraoperative quality assurance tools, such as completion angiography and/or transit time Doppler flowmetry have also been shown to have benefits to reducing technical errors which may improve early graft patency (21-23), although their benefit on clinical outcomes has not been consistently supported in clinical trials (6,24,25).

I. Choice of conduits – arterial versus saphenous vein

The choice of conduit for CABG has been shown to impact graft patency. The excellent early and late patency of *in situ* left internal thoracic arteries (LITA) (26,27) has stimulated the use of other arterial grafts, such as the right ITA and the radial artery (RA) (7,28-30). The ongoing Arterial Revascularization Trial, recruiting 3,000 patients, is the key RCT aiming to confirm the observational findings that bilateral ITA grafts purport improved clinical outcomes compared to a single ITA (31). Randomized trials, including one by our study investigators, have shown that the radial artery is as good or superior to a saphenous vein graft with respect to early and mid-term patency for CABG surgery (7,32,33). Total arterial revascularization with bilateral ITA and a RA is a well described contemporary CABG revascularization strategy aimed to accrue the aforementioned benefits. The Copenhagen Arterial Revascularization Randomization Patency and Outcomes Trial enrolled 331 patients to either bilateral ITA and a RA versus LITA and SVGs. Results at 1-year post-CABG showed equivalent clinical outcomes, with a tendency towards more early sternal healing complications in the total arterial cohort (34,35). However, at 14 months Muneretto et al show similar mortality, yet significantly lower rates of non-fatal MI, graft occlusion and recurrence of angina, in an elderly cohort of patients undergoing total arterial revascularization using a LITA and radial versus LITA plus SVGs (36,37).

Although still debated, the overall evidence from the literature suggests that that while two arterial grafts provide improved clinical outcomes compared to a sole LITA, a third arterial graft may not provide any significant benefit (34,35,38,39). Target coronary vessels/lesions are also limited for ITA and RA, and concerns regarding arterial graft spasm, string sign, graft site ischemia remain and poor wound healing including sternal infection (for bilateral ITAs) are notable (7,40-43). Furthermore, recently population-based studies indicate that multiple arterial grafting is used for only a quarter of patients undergoing isolated CABG (44). As such, ***SVGs remain an important conduit for patients undergoing CABG using a LITA or a LITA plus a second arterial graft***.

II. "No touch" technique of SVG harvesting

There are no systematic reviews concerning "no touch" SVG harvesting. A single Swedish cardiac surgical centre has published studies demonstrating that harvesting the SVG with a cushion of surrounding fat and without vein graft distension, the so-called "no touch" technique, significantly improved patency compared with conventional methods, i.e. stripping the vein of all adventitial tissue and distension with a balanced salt solution prior to implantation (45-47) (18 month patency: "no touch" 95% vs. conventional 89%, p=0.10; 8.5 year patency: "no touch" 90% vs. conventional 76%, p=0.01). By multivariate analysis, the "no touch" technique was strongly protective (OR 3.7, 95% CI 1.4-9.6, p=0.007). There is significant biological data from graft specimens that the "no touch" technique preserves vein architecture and nitric oxide synthase (47,48). The major limitation regarding the putatitive benefit of the "no touch" technique of vein harvesting is that the data has all been derived from a single centre. Replication of these results in multiple centres is required prior to the technique being widely adopted. However, the concept of avoiding trauma from SVG handling was also explored by a group in Bristol, UK, led by Angelini (49). They used a biodegradable external sheath to cover porcine SVG prior to use in a carotid bypass porcine model. While they found significant reductions in medial and intimal thickening in the SVGs, when applied in a phase I human trial (the Extent Study), results were not favourable. Conversely, post-hoc analysis from the PREVENT IV trial, and recent observational data published in NEJM, show that endoscopic harvesting of SVG results in significantly worse graft patency and clinical outcomes, likely due to increased trauma during harvesting and subsequent endothelial injury and dysfunction (5, 50). Overall there is clear equipoise to performing a multi-centre randomized clinical trial to validate the excellent single-centre results shown by Souza et al and determine whether the "no touch" technique is reproducible, feasible and generalizable (45-48).

III. The use of fish oil supplementation

Interest in fish oil supplementation originated following the initial discovery that dietary fish was associated with decreased cardiovascular risk in Greenland Eskimos (51). Several mechanisms have been postulated to explain the potential benefits of fish oils, including lipid lowering (52-55), plaque stabilization (56), anti-thrombotic (57), anti-inflammatory (58) or anti-arrhythmic properties (59-61). The American Heart Association recommends a daily intake of 1 gram daily, either in the form of fish or fish oil supplements as secondary prevention for patients with coronary artery disease (52). A meta-analysis, published earlier this year in BMJ, reviewed results from 12 studies totaling 32,779 patients (54). There was no significant improvement with fish oil supplementation for the endpoints of sudden death (OR 0.81, 95% CI 0.52 - 1.25) or all-cause mortality (OR 0.92, 95% CI 0.82 - 1.03) while cardiac deaths were reduced significantly (OR 0.80, 95% CI 0.69 - 0.92). The overview was heavily weighted by 2 studies, GISSI-Prevenzione (62,63) and JELIS primary and secondary prevention trials (64). The earlier post-MI DART trial in 2,033 men (not included in the meta-analysis) showed a 29% risk reduction in all-cause mortality in those with increased intake of fish-oils, with or without supplementation (65). Additional recent trials not included in the meta-analysis, GISSI-HF (66) and OMEGA (67) involving approximately 11,000 additional patients, revealed conflicting results. In the GISSI-HF trial, all-cause mortality was reduced 9% (adjusted HR 0.91, 95% CI 0.83 – 0.99) while benefits were not observed in OMEGA (ACC 2009, unpublished). Trials have consistently showed that for secondary prevention, the dose of at least 1g of fish-oil per day is required to accrue the most benefits (in an approximately 1.2:1 ratio of the active ingredients DHA:EPA) (55). Two studies have been completed in CABG patients. In a 160 patient RCT, fish oil supplementation started 5 days preoperatively was associated with a decreased incidence of postoperative atrial fibrillation (68). The role of fish oils on 1-year postoperative SVG patency was assessed in a 610 patient RCT by Eritsend et al (69). Fish oils were

protective for SVG patency (OR 0.77, 95% CI 0.60 - 0.99) and serum n-3 phospholipid levels were inversely correlated with graft occlusion. In addition to these potential gains, fish-oils are low-cost, usually well tolerated, and have been used in larger clinical trials in association with other evidence-based therapies (62,63,64,66,67).

IV. Risks to the safety of participants

There are no known additional risks associated with the study as compared to the usual standard of care (besides the inherent risks associated with CABG). Bypass surgery carries some risks, including a chance of heart damage (less than 5%) and death (less than 2%), stroke (about 5%). Although there are risks, the potential life-saving benefits of CABG surgery outweigh the risks. Additional complications associated with CABG surgery may include: bleeding, renal failure, infection, difficulty breathing, and/or arrhythmias (particularly atrial fibrillation). Both SVG harvesting techniques are used routinely in the centres participating in this study. The "no touch" technique may result longer harvesting times; however, there are no known risk associated with this. Damage to the saphenous nerve is inevitable when harvesting the SV by this technique; however, this also occurs frequently when using the conventional technique.

The most common side effect of taking fish oil supplements is a fishy aftertaste. This occurs in a moderate number of people. Gastrointestinal disturbance (which can include diarrhea, halitosis, eructation and nausea) is a low risk associated with taking fish oils. Taking fish oils with food rather than on an empty stomach has been shown to lower the risk of fishy aftertaste and GI disturbances. Some interactions may also occur between fish oil supplements and aspirin, other NSAIDS and herbs such as garlic and ginkgo. Such interactions might be manifested by increased susceptibility to bruising, nosebleeds, hemoptysis, hematemesis, or hematuria. Most who take fish oil supplements and the above drugs or herbs do not suffer from these problems. When they do occur, they are rare.

2 STUDY OBJECTIVES

The objective of the pilot phase of the study is to determine the number of eligible patients of the total isolated CABG patients, the consent rate, adherence to the protocol including rate of follow-up cardiac CT angiography, medication discontinuation and the overall incidence of the primary outcome. Achieving a recruitment rate of 1-2 patients/centre/month will determine if the study will proceed to the full phase III trial.

The primary objective of this study for the **Surgical Arm** is to determine whether a novel atraumatic ("no touch") technique of pedicled saphenous vein graft (SVG) harvesting results in a lower ***proportion of study SVGs*** that are totally (100%) occluded on 1-year post-CABG angiography and death due to cardiovascular or unknown causes, compared to conventional SVG harvesting techniques, in patients undergoing CABG.

The primary objective of this study for the **Pharmacological Arm** is to determine whether N-3 polyunsaturated fatty acid (fish oil) oral supplementation results in a lower ***proportion of patients*** with ≥1 graft (study SVG, non-study SVG or arterial graft) totally (100%) occluded at 1-year post-CABG angiography and death due to cardiovascular or unknown causes, compared to those who received placebo.

The secondary objectives for the **Surgical Arm** are to determine whether:

i. The "no touch" technique of SVG harvesting results in a lower ***proportion of study SVGs*** that have a significant stenosis (50-99%) on 1-year post-CABG angiography compared to conventional SVG harvesting techniques.
ii. The incidence and severity of adverse SVG harvesting events at 1-year post-CABG (infection, haematoma, swelling, neuropathy, quality of life measures) are similar between the "no touch" and conventional groups of patients.
iii. The incidence of the composite of non-fatal myocardial infarction (MI) (using new definition, see Section 5.5), all-cause mortality, and repeat revascularization (redo CABG or percutaneous coronary intervention [PCI]) perioperatively and at 1 year is lower in the "no touch" versus conventional group of patients.

The secondary objectives for the **Pharmacological Arm** are to determine whether:

iii. Fish oil supplementation results in a lower ***proportion of patients*** with ≥1 graft with a significant (50-99%) stenosis at 1-year post-CABG angiography, compared to those who received placebo.
iv. The incidence of the composite of non-fatal MI (using new definition, see Section 5.5), all-cause mortality, and repeat revascularization (redo CABG or PCI) perioperatively and at 1 year is lower in the fish oil versus placebo group of patients.

The tertiary objectives for the **Surgical Arm** are to determine whether the incidence of the composite of non-fatal myocardial infarction (MI) (using old definition, see Section 5.5), all-cause mortality, and repeat revascularization (redo CABG or PCI) perioperatively and at 1 year is lower in the "no touch" versus conventional group of patients.

The tertiary objectives for the **Pharmacological Arm** are to determine whether the incidence of the composite of non-fatal MI (using new definition, see Section 5.5), all-cause mortality, and repeat revascularization (redo CABG or PCI) perioperatively and at 1 year is lower in the fish oil versus placebo group of patients.

3 STUDY DESIGN

3.1 Type of study

This study is an International, multi-centre, prospective, controlled RCT using a 2x2 factorial design; involving a surgical intervention and a pharmacological intervention. For the surgical intervention, surgeons will be unblinded while patients, care providers, data collectors and outcome adjudicators will be blinded. For the pharmacologic intervention, patients, all care providers, data collectors and outcome adjudicators will be blinded. Approximately 10 Canadian and 40 International centers will participate.

3.2 Expected number of subjects

We expect to recruit 50 patients within the pilot phase. This is a convenience sample size to give us enough experience to assess our pilot objectives in support of a 1,550 patient study. The sample size for the full study is approximately 387 patients per group; for a total of 1,550 patients.

3.3 Method of treatment allocation

Prior to the trial, all surgeons will be provided with a copy of the trial protocol. Research personnel will screen patients in the preoperative assessment clinic to identify patients who fulfill the eligibility criteria. The personnel will also review the daily surgical list for eligible in-patients. Patients who fulfill the eligibility criteria will be approached for informed consent. Eligible and consenting patients will be randomized by calling a 24 hour interactive voice response system (IVRS) or by logging-in to a web-based randomization service. Treatment allocation to either "no touch" or conventional SVG harvesting, and fish-oil supplementation or placebo will be performed according to a computer generated randomization list. This will be stratified by site using a factorial design; blocked randomization will be performed with randomly sized blocks to maintain concealment. Randomization will also be stratified according to the control technique (open vs. endoscopic) to minimize bias following the pilot phase. Patients will be considered as randomized as soon as the treatment allocation is given over the telephone.

3.4 Duration of the study period for each subject

The surgical arm of the factorial design will involve an intervention which solely occurs intraoperatively having no impact on perioperative care. The pharmacological arm of the factorial design will involve patient compliance taking a medication from the time of consent (preoperative) to the time of follow-up at 1-year. Study supplements will be held the day of surgery and, following surgery, will commence once the patient is able to take oral medications. Patients will be followed for 1 year post-CABG.

4 STUDY POPULATION

4.1 Inclusion Criteria

i. Age >18 years
ii. Able to provide informed consent
iii. Isolated CABG, non-emergent, on- or off-pump (cardiopulmonary bypass)
iv. Primary or re-do CABG (if re-do, all previous grafts must be occluded)
v. Left ventricular ejection fraction >20%
vi. Require at least one SVG as part of revascularization strategy
vii. eGFR of at least 30 mL/min or higher

4.2 Exclusion Criteria

i. Unable to use greater SV due to previous vein stripping or poor quality on mandatory preoperative Duplex study and vein mapping
ii. Contraindication to receiving follow-up 64-slice cardiac CT angiography (allergy to contrast dye, renal failure with a creatinine >180 μmol/L, uncontrolled atrial fibrillation precluding proper gating of study)
iii. Pregnant women, or women of child-bearing age
iv. Allergy to fish oil/fish products, and non-medicinal ingredients of the study product (corn oil, soybean oil, gelatine, glycerol, or carob colouring)
v. Already taking fish oil supplements regularly (daily use in the past 30 days)
vi. Congenital or acquired coagulation disorders
vii. Patients considered to be of excessive risk of wound infection according to the **clinical judgment of the site surgical investigator**. N.B. Patients that are excluded for anticipated excessive risk of wound infection need to have the reasons for exclusion well documented in the screening log. We expect that the excluded patients will have advanced comorbidities or multiple risk factors for wound infection (such as peripheral vascular disease, peripheral edema, diabetes mellitus, obesity, advanced age, malnutrition, steroid dependence, concurrent infection, concurrent malignancy, or frailty).

5 STUDY PROCEDURES

5.1 Treatments

A. SURGICAL ARM

Eligible patients undergoing multi-vessel CABG with ≥1 SVG, will have the study SVG directed to the most important coronary target intended for a venous conduit. **In the experimental arm**, patients will be randomized to have the study SVG harvested from their lower leg using the novel, pedicled, atraumatic **"no touch" technique** (45-48). **In the control group**, patients will have their study SVG graft harvested in **a conventional fashion** without a pedicle.

The atraumatic "no touch" approach involves a longitudinal incision in the lower leg below the knee to the medial malleolus. However, the SVG is harvested with its surrounding tissue (pedicled). It is not manually dilated; it is left *in situ* until required and allowed to dilate when exposed to arterial pressure. All study patients will undergo duplex scanning and mapping of the saphenous veins preoperatively, to assess the quality of the vein as well as limit the creation of flaps during the vein dissection. Saphenous veins will be harvested from the thigh in the event that the lower leg vein is of poor quality or small size, or in patients with marked edema or peripheral vascular disease with concerns regarding wound healing.

The conventional approach involves a longitudinal incision in the lower leg, just below the knee to the medial malleolus. The vein is identified and the adventitial layer is stripped to directly expose and visualize the SV and its branches. Vein from the thigh may be harvested for similar reasons as

discussed above. It is then excised and distended using heparinized saline. It is standard practice in many institutions to harvest SV using an endoscopic, minimally invasive rather than an open approach, for reasons of improved cosmesis and because local wound complications are reduced (70). For relevance and to reflect contemporary trends in practice, the control group/conventional approach may include the minimally harvested SVG, which is otherwise prepared in a similar manner, i.e. stripped of adventitia and manually distended, albeit through a smaller incision. A recent observational study of a large multi-centre sample (50) and post hoc secondary analysis from the large multi-centre PREVENT IV RCT (5) reported poorer patency and clinical outcomes using endoscopically harvested SVG, however, institutions that predominantly employ endoscopic harvesting may continue to choose this technique.

All patients will have their left anterior descending coronary artery grafted with an internal thoracic artery, the standard of care for CABG. The study SVG, directed to the most important target intended for a venous conduit, may be to the second, third or fourth territory chosen; this design allows for the use of multiple arterial grafting as per surgeon discretion/patient indication. The surgeon will identify the proposed target for the study graft prior to surgery. While the site of SVG harvesting (right or left leg) and the coronary target grafted (circumflex, right coronary, or branch [i.e. diagonal]) will not be randomized, all site and target vessel characteristics will be recorded for post-hoc multivariable regression analysis. Surgeons will be encouraged to use the study SVG for a single distal target. If a sequential graft is required, it will only be considered patent at follow-up angiography if it is entirely patent to all sequentially grafted targets. If more than one SVG is used, surgeons will also be encouraged to harvest all SVGs using the same technique (either all "no touch" or all conventional). While this decision will not impact the primary outcome of 'study graft' patency between the two groups (i.e. the SVG directed to the most important vein graft target), it may increase the ability to detect differences in clinical outcomes at 1-year.

B. PHARMACOLOGICAL ARM

Patients will be randomized to receive either fish-oil oral supplements (Ocean Nutrition, Dartmouth, NS) or a colour and taste-matched placebo (Ocean Nutrition, Dartmouth, NS). Upon randomization at the time of accepting referral for surgery and consent, the subjects will consume two 1g tablets, daily (as used in prior clinical trials) for 1 year. Study supplements will not be administered on the day of surgery. The duration of preoperative therapy is not mandated but will be evaluated post hoc; surgery should occur within 30 days of randomization. In the event that the patient misses any dose(s) of study supplement, patients will be instructed to resume the next scheduled study dose and discouraged from taking more than two study supplements per day.

Postoperative medical management using aspirin and/or other antithrombotic agents, statins, beta blockers and/or ACE-inhibitors will be prescribed in a standardized fashion according to published STS and AHA/ACC guidelines.

5.2 Schedule of visits and observations

Study subjects will have clinical assessments preoperatively, pre-discharge, 1-month, 3-month, 6-month, 9-month, and 1-year postoperatively (see Table 1 and Figure 1). The preoperative visit will consist of an evaluation of eligibility criteria, obtaining informed consent, gathering demographical, medical history, blood work and ECG information. Fish oil supplementation or placebo will be

commenced at this visit. Study supplements will not be administered on the day of surgery. Blood will be drawn at induction for the preoperative CK-MB (5 mL draw for preoperative CK-MB). The discharge visit will consist of a patient evaluation and blood work (5 mL draw for 24 hour post-operative CK-MB). At this time, a 3-month supply of the fish oil supplement or placebo will be given (by the Site Study Nurse). The 30-day visit will consist of a patient evaluation, assessment of leg wound, and a drug compliance check. The 3-month visit will consist of a patient evaluation, assessment of leg wound, and drug compliance check. The 6-month and 9-month visits will consist of a patient evaluation and drug compliance check. At the 3-month, 6-month and 9-months visits, a 3-month supply of the fish oil supplement or placebo will be given. The 1-year visit will consist of a patient evaluation, blood work (5 mL draw for serum creatinine), assessment of leg wound, drug compliance check, and cardiac CT angiography (acceptable window for angiography is 9-15 months postoperatively). ECG and blood work (creatinine) information will also be collected at this time as the patient will need to have these tests done prior to undergoing the CCTA. Patients with an estimated GFR < 30 mL/min will not be eligible for the study CT angiography. Patients with an estimated GFR of 30-60 mL/min may undergo the study CT angiography with renal precautions according to the institutional protocol, which may include any or all of the following: saline hydration, sodium bicarbonate loading, n-acetyl cysteine prophylaxis, and temporary stoppage of potentially nephrotoxic drugs and metformin.

Table 1: Schedule of follow-up

VISIT SCHEDULE	Pre-op	OR Day	Discharge	30 Day	3 Mth	6 Mth	9 Mth	1 Year
Evaluation of Inclusion Criteria	✓							
Informed Consent	✓							
Demographics	✓							
Blood Work (creatinine)	✓		✓[a]					✓
Blood Work (CK-MB)	✓		✓[b]					
Medical History	✓							
Start Fish Oil Supplementation/Placebo	✓[c]							
Details of Surgical Procedure		✓						
Patient Evaluation	✓		✓	✓	✓	✓	✓	✓
Clinical Outcomes			✓	✓	✓	✓	✓	✓
Leg Wound Assessment				✓	✓			✓
Refills Given of Supplement/Placebo			✓		✓	✓	✓	
Supplement Compliance Checks				✓	✓	✓	✓	✓
ECG	✓		✓[d]					✓
Quality of Life Questionnaire	✓			✓				✓
Cardiac CT Angiography								✓

[a] Highest serum creatinine value within seven days post surgery and last serum creatinine value prior to hospital discharge
[b] CK-MB at 24-hours postoperatively
[c] Study supplements not to be administered on day of surgery
[d] ECG 24-hours postoperatively and within 4-7 days postoperatively

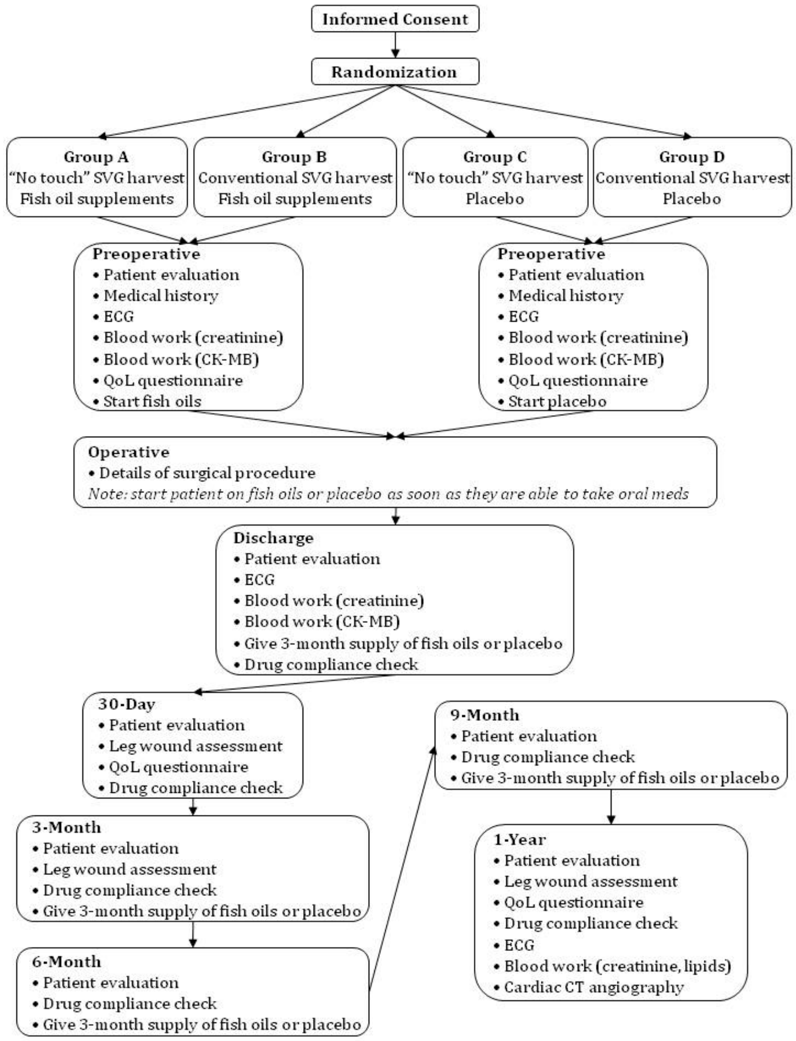

Figure 1: Flow chart of scheduled patient visits

5.3 Selection procedures (entry procedures)

Written informed consent for the study must be obtained prior to randomization. Key baseline patient characteristics will be recorded on the Case Report Forms (CRFs). Results of tests to confirm inclusion/exclusion criteria will be maintained in the patient files. Treatment allocation will be performed as stated above (see 3.3). Baseline CRFs should be completed soon after randomization.

5.4 Encouraging compliance

Noncompliance is not expected to be a major issue in this trial. The interventions are simple and the surgical intervention occurs at a single time-point and is controlled by the treating physician,

outcomes are objective, and data-forms are concise. Patient compliance to the study supplements will be monitored at follow-up visits via pill counts.

Patients are generally followed by their surgeons/cardiologists for several years after their procedure, and it is expected 99.9% follow-up at one month and over 99% follow-up at 6 months will be obtained. PHRI has substantial experience in documenting vital status in >99.5% in its clinical trials involving over 200,000 subjects recruited from over 50 countries and followed for up to 8 years. This expectation will be met in this study, especially with a post-surgical population in whom clinical follow-up by the surgeon for a minimum of 3 months is generally routine.

The overall study has been designed such that only one SVG is required to facilitate recruitment. We did consider restricting the study to patients who required at least 2 SVGs, as the event rates would presumably be greater. The chosen protocol does allow patients who are having only 2 grafts constructed (ITA and 1 SVG) and is compatible with patients undergoing more extensive arterial revascularization. We anticipate that the average number of vein grafts/patient will be 1.5 (as in GRIIP) (6). We considered restricting the control group to open harvesting rather than allow for endoscopic harvesting to be performed as well. We thought it important to include endoscopic harvesting as it is a commonly used technique, and there is recent evidence that endoscopic vein harvesting purports worse patency and clinical outcomes than conventional SVG harvesting (50). Although endoscopic patients will usually be unblinded, endpoint adjudication is blinded. Our post-hoc analysis will also aim to elucidate any difference in graft patency in the control SVGs harvested in the conventional open versus endoscopic techniques. We expect that the RRR will be similar or greater comparing endoscopic and no-touch versus open control and no-touch.

The operator dependence of the “no touch” technique, along with endoscopic vein harvesting at particular institutions, may impact on the outcomes determined. A multi-centre approach should allow for this to become evident and allow for the valid conclusion about the ability to generalize the intervention, irrespective of its benefits.

5.5 Study measurements

Study Outcomes

Primary Outcome

Surgical Intervention: The ***proportion of study SVGs*** (“no touch” versus conventionally harvested) which are totally (100%) occluded on 64-slice cardiac CT angiography at 1-year post-CABG and death due to cardiovascular or unknown causes.

Pharmacological Intervention: The ***proportion of patients*** with ≥1 graft totally (100%) occluded on 64-slice cardiac CT angiography at 1-year post-CABG and death due to cardiovascular or unknown causes, comparing the fish-oil to placebo groups. Fish oils can theoretically affect patency of any of the bypass grafts (study or non-study, saphenous veins or arterial), although the effect would presumably be greatest in saphenous veins. We have therefore defined the primary outcome

for the pharmacologic intervention as the proportion of patients with at least 1 graft occluded (saphenous or arterial).

Any clinically directed angiogram (CTA or conventional) occurring up to 15 months postoperatively will contribute to the primary endpoint. Patients with poorly controlled atrial fibrillation or claustrophobia will be approached for conventional angiography. We anticipate from past experience that this will apply to <2% of patients.

Secondary Outcomes

Surgical Intervention: The two groups will be compared with respect to:

i. The number of study SVGs with a significant (50-99%) stenosis on 1-year CTA.
ii. The number of study SVGs with a significant stenosis or total occlusion at 1-year CTA.
iii. The incidence of and severity of adverse SV harvesting events by 1-year (infection, haematoma, swelling, neuropathy).
iv. The incidence of the composite endpoint of perioperative and 1-year non-fatal MI (using new definition, see "Early perioperative myocardial infarction (within 72 hours of surgery)" below), all-cause mortality, repeat revascularization (redo CABG or PCI) (major adverse cardiac events, MACE) and stroke.

Pharmacological Intervention: The two groups will be compared with respect to:

i. The proportion of patients with ≥1 graft with a significant (50-99%) stenosis on 1-year CTA.
ii. The proportion of patients with ≥1 graft with a significant stenosis or total occlusion at 1-year CTA.
iii. The proportion of venous and arterial grafts considered separately with total occlusion and/or stenosis, defined above.
iv. The incidence of the composite endpoint of perioperative and 1-year non-fatal MI (using new definition (see "Early perioperative myocardial infarction (within 72 hours of surgery)" below), death, repeat revascularization (redo CABG or PCI) (major adverse cardiac events, MACE) and stroke.

NB: Graft occlusion determines the primary endpoint and graft stenosis, the secondary endpoint, as graft occlusion is generally of greater clinical importance than stenosis. Graft occlusion is the primary mode of SVG failure 1 year postoperatively (7).

Tertiary Outcomes

Surgical Intervention: The two groups will be compared with respect to the incidence of the composite endpoint of perioperative and 1-year non-fatal MI (using old definition, see below), all-cause mortality, repeat revascularization (redo CABG or PCI) (major adverse cardiac events, MACE) and stroke.

Pharmacological Intervention: The two groups will be compared with respect to the incidence of the composite endpoint of perioperative and 1-year non-fatal and fatal MI (using old definition, see

below), death, repeat revascularization (redo CABG or PCI) (major adverse cardiac events, MACE) and stroke.

Definitions of Study Outcomes

Cardiovascular (CV) death: All deaths in the first 30 days are considered to be CV deaths. All deaths after the first 30 days are considered CV deaths unless a specific non-cardiovascular cause is evident and considered to be the cause of death (e.g. malignancy). Furthermore, patients who die during the index hospitalization but after the initial 30 days period (i.e. long ICU stay with sepsis) will be considered as CV deaths.

Myocardial infarction: The pathophysiology of myocardial injury sustained perioperatively is likely different from that when injury is sustained later. Early injury can be a manifestation of either graft occlusion, or of a more general insult secondary to ischemia reperfusion, inflammation, and coronary embolization or spasm. Late injury is likely much more similar to the traditional acute coronary syndrome/atherosclerotic process with native vessel or graft occlusion. The definitions for these events differ as below:

(a) Early perioperative myocardial infarction (within 72 hours of surgery):

i. New Definition:

1) A CK-MB measurement ≥ 5 times the upper limit of normal with new pathological Q waves or new LBBB (Q wave MI) **or** without new pathological Q waves or new LBBB (non-Q wave MI); or
2) Angiographic evidence of graft occlusion or native coronary artery occlusion; or
3) Imaging evidence of new loss of viable myocardium.

ii. Old Definition: Early MI is defined as CK-MB ≥ 70 ng/mL and ECG changes consistent with myocardial injury or CK-MB ≥ 100 ng/mL in all patients.

(b) Late perioperative myocardial infarction (later than 72 hours after surgery): ECG changes consistent with myocardial infarction (new significant Q waves in two contiguous leads) or evolving ST-segment or T-wave changes in two contiguous leads signifying ischemia or new left bundle branch block or ST segment elevation and elevated cardiac markers (troponins or CK-MB) in the necrosis range. Myocardial injury occurring after a PCI are included in the late perioperative Myocardial Injury group but are defined as elevation of cardiac markers ≥ 3 times upper limit of normal within 24 hours of PCI or characteristic evolution of new ECG changes.

New atrial fibrillation: New onset atrial fibrillation requiring institution of pharmacologic therapy or cardioversion.

Transfusion requirements: Autologous blood, homologous processed RBC, whole blood, plasma, platelets, cryoprecipitate will be tracked.

12 hour chest tube output: Total chest tube output in the first 12 hours or until the tubes are removed, whichever comes earlier.

Duration of mechanical ventilation: Time from ICU admission to first extubation in hours.

Length of ICU stay and hospital stay: Time from post-operative ICU admission to ICU discharge (hours) and hospital discharge (days).

Stroke: Diagnosis of stroke will require focal neurological symptoms with rapid onset, lasting at least 24 hours. It is strongly recommended (but not required) that an imaging procedure such as a CT scan or MRI be performed. All strokes will be classified as definite ischemic, definite hemorrhagic or type uncertain. A vascular imaging procedure such as a carotid ultrasound is recommended whenever possible (but not required) for subclassification of ischemic strokes into cardioembolic, lacunar or large artery.

Surgical Site Infection: Surgical site infection is defined as an infection that arises within 30 days of an operative procedure and at the site of surgical intervention. Symptoms and signs suggestive of a surgical site infection include wound erythema and blanching, tenderness, pain, purulent discharge, fever (temperature >38.0 C), and leukocytosis. A superficial surgical site infection involves the skin or subcutaneous tissues alone, whereas a deep surgical infection involves the fascia or muscle layers, and an organ space surgical site infection involves the deeper anatomic areas operated during the surgical procedure. JV: The temporal window for the definition of sternal infection is 12 months. I don't know about leg or arm, but I think we should use 3 or 12 months.

Wound complications:

Deep mediastinal wound infection: Surgical site infection requiring surgery

Superficial wound infection: Surgical site infection not requiring surgery

Sterile wound dehiscence: Culture negative dehiscence of a wound

Major Bleed: Major bleeding that is defined as bleeding greater than 48 hours post-operative and associated with any of the following: death, drop in hemoglobin of at least 2 g/dL, significant hypotension with the need for inotropic agents, bleeding requiring surgical intervention [other than vascular site repair], intracranial hemorrhage, intraocular hemorrhage (excluding subconjunctival hemorrhage), or the requirement for a transfusion of at least 2 U of blood.

Renal failure: Requirement for renal replacement therapy (e.g., dialysis, continuous hemofiltration, renal transplant). Hemofiltration or dialysis only during cardio-pulmonary bypass does not constitute a requirement for renal replacement therapy. Patients who receive dialysis within 1 month prior to the surgery are not eligible for this endpoint.

Repeated coronary revascularization: New CABG procedure or PCI associated with documented ischemia by ECG *and* graft failure or new culprit lesion (≥ 70% luminal stenosis).

Recurrence of angina: New onset of typical chest angina with documented ischemia by stress testing (ECG, echocardiography, or nuclear) or persistence of CCS grade ≥2 angina after the surgery.

6 SUPPLEMENT SUPPLIES

6.1 Study Supplements

Patients will receive either fish oil supplements or placebo to be taken twice daily for 1 year post-CABG. Patients will start taking the supplements as soon as they consent and are randomized until their surgery (study supplements not to be administered on day of surgery), and will commence again postoperatively as soon as they are able to take oral medications. The fish oil supplement (Omega Pure 40/20 EE 1000 mg capsules – containing 340 mg eicosapentaenoic acid (EHA) and 170 mg docosahexaenoic acid (DHA)) and placebo (50/50 corn/soybean placebo 1000 mg capsules) will be obtained from Ocean Nutrition Canada. Fish oils or placebo will be given to the patient by the Study Nurse. Patients will receive a bottle containing 185 pills (enough for 3 months) when they are discharged, another bottle at the 3, 6 and 9 month follow-up visits.

6.2 Packaging and labeling

The fish oil supplement and placebo capsules will be packaged, labeled and shipped to the participating sites by Bay Area Research Logistics in Hamilton. The bottles will be labeled with a treatment ID number so as to maintain blinding.

6.3 Storage conditions

Both fish oil supplements and placebo should be stored at room temperature. There are no specific transportation conditions and no special handling requirements. The fish oil supplements and placebo have a shelf life of 3 years.

6.4 Responsibilities

All supplement supplies that will be used in the study must be maintained securely, under the responsibility of the Investigator, or other personnel allowed to store and dispense the supplements, according to the national regulations.

It is the Investigator's responsibility to ensure that an accurate record of supplements issued and returned is maintained.

Under no circumstances will the investigator supply study supplements to a third party, allow the study supplements to be used other than as directed by this protocol, or destroy or dispose of the supplements in any other manner.

7 SUBJECT SAFETY

7.1 Monitoring of adverse events

An ***Adverse Event*** (AE) is any untoward medical occurrence in a patient or clinical investigation subject who has received treatment; it does not necessarily have to have a causal relationship with this treatment.

A ***Serious Adverse Event*** (SAE) is any untoward medical occurrence which:

- Results in death, or
- Is life-threatening, or
- Requires in-patient hospitalization or prolongation of existing hospitalization, or
- Is a congenital anomaly/birth defect

The term "life-threatening" in the definition of "serious" refers to an event in which the patient was at risk of death at the time of the event; it does not refer to any event which hypothetically might have caused death if it was more severe. Medical and scientific judgment should be exercised in deciding whether other important medical events should be considered serious. Expected disease-related events/outcomes, other study outcomes or study procedural-related events will not be reported as SAEs but will be reported on specific outcome CRFs. Adverse events will be followed until either resolution or baseline status is achieved.

7.2 Safety instructions specific to the trial

Patients must immediately contact the investigator in the event of unexplained symptoms or effects that may occur during or after the trial.

Serious adverse reactions can be "expected" (i.e. the safety outcomes that have been pre-defined) or "unexpected" if they would not be expected among perioperative patients given standard therapies and not previously described with fish oil supplementation. Previously described serious adverse reactions that are part of the clinical course of patients should be recorded on the case report forms. All serious adverse reactions, both expected and unexpected, need to be reported immediately (i.e. within 24 hours of knowledge of the event) to the Project Office by completing the appropriate Case Report Form. The Project Office will report all serious adverse reactions within Canada to Health Canada's Natural Health Products Directorate (NHPD). If the serious adverse reaction is fatal or life threatening, the Project Office will notify the NHPD no later than seven days after knowledge of the event. If the serious adverse reaction is neither fatal nor life threatening, the Project Office will notify the NHPD no later than 15 days after knowledge of the event.

Emergency Unblinding

Legitimate situations such as an adverse drug reaction may require unblinding. We recommend that all unblinding decisions be made jointly with the Project Office. The principal investigator should approve the request for unblinding wherever possible. If the local study investigator believes emergency unblinding is essential for the patient's management then it can be undertaken either by following the instructions provided in the Manual of Operations.

7.3 Premature Withdrawal/Discontinuation Criteria

There are no known serious adverse effects of taking fish oil supplements. The most common side effect is gastrointestinal (GI) intolerance. If a study participant experiences severe GI intolerance which is suspected to be due to the study supplements, the following steps will be taken: 1) dose reduction – patient will only take 1 pill per day (instead of 2) for 2 weeks to see if this improves symptoms; 2) if symptoms persist, supplements will be discontinued for 2 weeks; 3) if symptoms improve, patient will be started on the 1 pill per day regime to see if tolerated; 4) if patient tolerates this treatment, full dosing will resume; 5) if symptoms reappear, patient will be permanently discontinued from the supplement. Patients will remain blinded, unless absolutely necessary.

8 STATISTICAL CONSIDERATIONS

8.1 Study outcomes

The aim of this study for the **Surgical Arm** is to determine whether the "no touch" technique results in a lower proportion of study SVGs that are totally (100%) occluded on 1-year post-CABG angiography, compared to conventional SVG harvesting techniques, and death due to cardiovascular or unknown causes. The aim for this study for the **Pharmacological Arm** is to determine whether N-3 polyunsaturated fatty acid (fish oil) oral supplementation results in a lower proportion of patients with ≥1 graft (study SVG, non-study SVG or arterial graft) totally (100%) occluded at 1-year post-CABG angiography, compared to those who received placebo. These events will be adjudicated, as defined in the operations manual.

8.2 Analysis population

The main analysis will be by an intention-to-treat approach (i.e., all randomized patients in the study will be included in the analysis). At least 1,550 patients will be equally allocated to the four treatment groups.

8.3 Statistical Methods

The primary comparison for the 2 main study questions ("no touch" vs. conventional SVG harvesting [primary endpoint: study SVG occlusion]; fish oil supplementation vs. placebo [primary endpoint: proportion of patients with >1 graft occluded]) will be performed using a Chi-square test according to the intention-to-treat principle. A test for interaction will be tested similarly and with logistic regression. Statistical significance is assumed for $p<0.05$. The secondary graft occlusion endpoints, graft stenosis, or graft occlusion or significant stenosis, will also be tested with Chi-square or Fisher's tests where appropriate, considering graft stenosis as a dichotomous variable, as well as logistic regression treating graft stenosis as an ordinal variable. Additional modeling will be performed post hoc with logistic regression, generalized estimating equation and bootstrapping techniques, to evaluate covariates of graft occlusion (7,18,71). Variables of interest in addition to the usual demographic factors include: open vs. endoscopic vein harvesting, serum lipid values; study centre; surgeon; time period of patient entry (to address a possible learning effect); timing of postoperative angiography; parameters related to the distal coronary artery target such as, size, quality, and factors related to the specific coronary artery grafted including the severity of the proximal stenosis. Other categorical outcomes (major cardiac events and vein harvest site complications) will also be compared with either Chi-square or Fisher's Exact test where appropriate. The harvest site infection scores will be tested using t tests.

8.4 DSMB and interim analysis

The independent Data Safety Monitoring Board (DSMB) will ensure patient safety, receive and review interim analyses of efficacy data, provide feedback to the Steering Committee, and ensure the study follows the highest standards of ethics. The DSMB will consist of a cardiac surgeon, cardiologist and epidemiologist not affiliated with this trial. The DSMB will follow clinical events (all-cause mortality, perioperative MI, low-output syndrome, vein harvest site infections, drug adverse events/intolerance) for both the surgical and pharmacologic arms. A single interim analysis will be performed following completion of 50% of angiograms. The DSMB will recommend stopping the trial if there is an excess of MACE or if the rate of graft occlusion in a treatment group is greater than the rate of graft occlusion in a control group, with a significance of $p <0.001$. As the design is factorial, one treatment modality could be stopped if the difference between groups is highly significant ($p<0.001$), and the other arm continue. A single interim analysis will be performed following completion of 50% of the follow-up angiograms, using a p value of <0.001 to preserve the 0.05 alpha level. The final analysis will be performed following completion of all angiograms. A decision to continue or stop the trial would be based on a number of factors in addition to the statistical significance of the main results, including consistency of the pattern of the data over time and an assessment of net benefit-risk ratios. At any time during the study, if safety concerns arise the DSMB chairperson will assemble a meeting of the full committee. The DSMB will make their recommendations to the steering committee after considering all the available data and any external data from relevant studies.

8.5 Sample size calculation

A. SURGICAL ARM

The sample size estimation is based on the current prevalence of 1-year SVG occlusion using the conventional harvesting technique to be 20% (5,6). We consider a 30% relative risk reduction (RRR) in graft occlusion using the "no touch" technique to be a clinically important difference, consistent with RRRs reported for previous interventions to improve graft patency (antiplatelet therapy, use of statins post CABG, radial versus saphenous veins) and very conservative in terms of Souza's clinical trial results (45,46). A sample size of 615 patients in each group will provide 80% power for a 2-tailed alpha of 0.05, to identify a RRR of 30% (20% occlusion rate in the control/ conventional harvesting group, to 14% in the experimental/"no touch" group – see Table 2a).

B. PHARMACOLOGICAL ARM

The literature suggests that fish-oils provide a RRR of about 25% compared to placebo (54,55, 65,69). Given a two-tailed alpha of 0.05, and an estimated 30% of control patients with ≥1 graft occluded at 1-year, a sample of 540 patients in each group (fish-oil versus placebo) will provide a power of 80% to detect a RRR of 25%. A sample size of 615 patients (required for the surgical intervention) provides approximately 85% power to test the fish oil hypothesis (see Table 2b).

We anticipate that 80% of patients will undergo 1-year coronary CT angiography, which is consistent with our past trial experience (7). The primary reason that patients failed to undergo late angiography in RAPS was late withdrawal of consent in 77 patients, protocol violation in 17 patients, new contraindication precluding research angiography in 19 patients and death in 8 patients (7). To adjust for this potential loss to follow-up angiography, the corrected sample size is 769 patients in each surgical group to ensure adequate statistical power when making our conclusions for both arms of the factorial design. Thus we aim to recruit a **total of 1,550** patients for the overall study across ~50 participating sites, 10 Canadian and the remaining International. The pilot phase will recruit 50 patients from 10 sites (5 Canadian and 5 International). Pilot patients will be included in the final analysis. *While MACE between groups will be tested using the total sample size, we will only be able to detect very large differences at 1 year, as MACE is expected to be approximately 10 - 12%* (6,7,17).

Table 2a: Sample Size Estimates for Primary Endpoint (Surgical Arm) – 1-Year Graft Occlusion (two-tailed α=0.05, β=0.20)

**Control Event Rate**	**Relative Risk Reduction**									
	**20%**		**25%**		**30%**		**35%**		**40%**	
	**No-Touch Event Rate**	**Sample Size**	**No-Touch Event Rate**	**Sample Size**	**No-Touch Event Rate**	**Sample Size**	**No-Touch Event Rate**	**Sample Size**	**No-Touch Event Rate**	**Sample Size**
**15%**	12.0%	**2036**	11.25%	**1272**	10.5%	**862**	9.75%	**617**	9.0%	**460**
**16%**	12.8%	**1889**	12.0%	**1181**	11.2%	**800**	10.4%	**573**	9.6%	**427**
**17%**	13.6%	**1759**	12.75%	**1100**	11.9%	**745**	11.05%	**534**	10.2%	**398**
**18%**	14.4%	**1644**	13.5%	**1028**	12.6%	**697**	11.7%	**499**	10.8%	**373**
**19%**	15.2%	**1540**	14.25%	**964**	13.3%	**654**	12.35%	**469**	11.4%	**350**
**20%**	16.0%	**1447**	15.0%	**906**	14.0%	**615**	13.0%	**441**	12.0%	**329**
**21%**	16.8%	**1363**	15.75%	**854**	14.7%	**579**	13.65%	**416**	12.6%	**310**

**22%**	17.6%	**1287**	16.5%	**806**	15.4%	**547**	14.3%	**393**	13.2%	**293**
**23%**	18.4%	**1217**	17.25%	**763**	16.1%	**518**	14.95%	**372**	13.8%	**278**
**24%**	19.2%	**1153**	18.0%	**723**	16.8%	**491**	15.6%	**353**	14.4%	**264**
**25%**	20.0%	**1094**	18.75%	**686**	17.5%	**466**	16.25%	**335**	15.0%	**250**

The shaded area represents the range of event rates and risk reductions that will be achieved given the targeted enrolment of 615 patients required for follow-up angiography, to achieve power of 80% in the surgical arm (two-tailed alpha of 0.05).

Table 2b: Sample Size Estimates for Primary Endpoint (Pharmacological Arm) – 1-Year Graft Occlusion (two-tailed α=0.05, β=0.20)

**Control Event Rate**	**Relative Risk Reduction**									
	**15%**		**20%**		**25%**		**30%**		**35%**	
	**Fish Oils Event Rate**	**Sample Size**	**Fish Oils Event Rate**	**Sample Size**	**Fish Oils Event Rate**	**Sample Size**	**Fish Oils Event Rate**	**Sample Size**	**Fish Oils Event Rate**	**Sample Size**
**20%**	17.0%	**2629**	16.0%	**1447**	15.0%	**906**	14.0%	**615**	13.0%	**441***
**22%**	18.7%	**2336**	17.6%	**1287**	16.5%	**806**	15.4%	**547**	14.3%	**393***
**24%**	20.4%	**2091**	19.2%	**1153**	18.0%	**723**	16.8%	**491***	15.6%	**353***
**26%**	22.1%	**1884**	20.8%	**1040**	19.5%	**652**	18.2%	**444***	16.9%	**319***
**28%**	23.8%	**1707**	22.4%	**943**	21.0%	**592**	19.6%	**403***	18.2%	**290***
**30%**	25.5%	**1554**	24.0%	**859**	22.5%	**540***	21.0%	**367***	19.5%	**264***
**32%**	27.2%	**1419**	25.6%	**785**	24.0%	**494***	22.4%	**337***	20.8%	**242***
**34%**	28.9%	**1300**	27.2%	**720**	25.5%	**453***	23.8%	**309***	22.1%	**223***
**36%**	30.6%	**1195**	28.8%	**663**	27.0%	**417***	25.2%	**285***	23.4%	**206***
**38%**	32.3%	**1101**	30.4%	**611**	28.5%	**385***	26.6%	**263***	24.7%	**190***
**40%**	34.0%	**1016**	32.0%	**564**	30.0%	**356***	28.0%	**244***	26.0%	**176***

The sample sizes noted with an asterix (*) represents the range of event rates and risk reductions that will be achieved for the pharmacological arm, given the targeted enrolment of 540 patients required for follow-up to achieve power of 80% (two-tailed alpha of 0.05). The sample sizes included in the shaded region represent the range of event rates and risk reductions that will be achieved for the pharmacological arm, given enrolment of 615 patients/arm planned for the surgical arm.

9 STUDY ORGANIZATION

9.1 Coordinating Centre

The trial will be conducted internationally and coordinated at the Population Health Research Institute (PHRI) at McMaster University and Hamilton Health Sciences, Hamilton Canada.

9.2 National Coordinators

Within each country, there will be a National Coordinating Office headed by an expert in cardiovascular disease and/or clinical trials. These units will be responsible for obtaining the national regulatory approvals, coordination of research ethics application at each site, organization

of training and follow-up meetings, dealing with other issues related to recruitment, data quality, and ensuring high rates of follow-up. All the National Coordinators along with the Operations Committee will serve on the Study Steering Committee, which will meet at least every 3 to 4 months by telephone or in person. The Steering Committee will be consulted regarding major decisions about the study.

9.3 Event Adjudication Committee

The Events Adjudication Committee will blindly evaluate all primary outcome events using standardized definitions, along with supporting documentation. Members of the Events Adjudication Committee will be chosen based on their clinical expertise. All event adjudication will be blinded to treatment group.

10 ETHICAL AND REGULATORY STANDARDS

10.1 Regulatory and ethical considerations

Prior to initiation of a study site, the Study Sponsor, will obtain approval from the appropriate regulatory agency to conduct the study in accordance with applicable country-specific regulatory requirements. The study will be conducted in accordance with all applicable regulatory requirements.

The study will be conducted in accordance with Good Clinical Practice (GCP), all applicable subject privacy requirements, and the guiding principles of the declaration of Helsinki, including, but not limited to:

- Institutional Review Board (IRB)/Independent Ethics Committee (IEC) review and approval of study protocol and any subsequent amendments.
- Subject informed consent.
- Investigator reporting requirements.

10.2 Laws and regulations

This study will be conducted in accordance with laws and regulations of the countries in which the trial is performed, as well as any applicable guidelines.

10.3 Informed consent

It is the responsibility of the Investigator to obtain informed consent in compliance with national requirements from each subject prior to entering the trial or, where relevant, prior to evaluating the subject's suitability for the study.

The informed consent document used by the Investigator for obtaining subject's informed consent must be reviewed and approved by PHRI prior to Ethics Review Committee or similar body (IRB, REB) submission.

10.4 Ethics Review Committee

The Investigator must submit this protocol to an Ethics Review Committee or a similar body (IRB, REB) and is required to forward a copy of the written approval/advice signed by the Chairman to the Sponsor.

On the approval/advice sheet, the trial (SUPERIOR SVG – 2011-06-13), the study documents (protocol and informed consent material) and the date of the review should be clearly stated.

Drug supplies will not be released and the trial will not start until a copy of this written approval/advice has been received by PHRI.

11 STUDY MONITORING

11.1 Responsibilities of the investigator(s)

The Investigator(s) undertake(s) to perform the study in accordance with Good Clinical Practice. The Investigator is required to ensure compliance with respect to the investigational drug schedule, visit schedule and procedures required by the protocol. The Investigator agrees to provide all information requested included in the Case Report Forms in an accurate and timely manner according to instructions provided.

11.2 Data collection on case report forms (CRFs)

It is the responsibility of the Investigator to prepare and maintain adequate and accurate CRFs which have been provided by the study to record all observations and other data pertinent to the clinical investigation. All CRFs should be completed in their entirety in a timely fashion.

Completed CRFs will be transmitted through the iDataFax system used by PHRI runs automatic checks on the accuracy and completeness of data. iDataFax allows for documentation of all transmitted case report forms as both data and images. All received data as well as quality control reports are stored in electronic format. When necessary, telephone communication with the sites either directly or through the Site Coordinators would be used. Data will be housed at each center in password protected computers and data collection forms will be stored in locked file cabinets within a locked room accessible only to site research coordinators. Data entered on computer files

will be done using unique identifiers instead of patient names or hospital file number, in order to assure patient privacy. Files will be transmitted between sites using unique identifiers only. They will occur via encrypted memory stick devices or via secure online website. Data records will be maintained for at least 15 years.

12 ADMINISTRATIVE RULES

12.1 Curriculum vitae

An updated copy of the curriculum vitae for each Investigator and co-Investigator will be provided to the Sponsor prior to the beginning of the study.

12.2 Record retention in investigating centre(s)

The Investigator must maintain all study records, patient files and other source data for the maximum period of time permitted by the hospital, institution or private practice and must be consistent with national regulatory requirements and ICH guidelines. It is recommended that the Investigator retain the study documents at least fifteen (15) years after the completion or discontinuation of the Clinical Trial. However, applicable regulatory requirements should be taken into account in the event that a longer period is required.

13 OWNERSHIP OF DATA AND USE OF THE STUDY RESULTS

The PHRI Project Office and the Steering Committee of the study have the ownership of all data and results collected during this study. In consequence, the PHRI Project Office reserves the right to use the data of the present study, either in the form of CRFs (or copies of these), or in the form of a report, with or without comments and with or without analysis, in order to submit them to the Health Authorities of any country. The Steering Committee has full rights to publication based on data from this study, without restriction.

14 PUBLICATIONS

All analyses for publication will be provided by the PHRI Project Office. The responsibility for presentations and/or publications belongs to the Steering Committee. The final content of the manuscript is the responsibility of the Steering Committee.

Publication of the main findings of this study will be made jointly in the name of all wholehearted collaborators. Other papers will be authored based on the contributions of the individuals to the overall study. All the trial participants (Investigators and committee members) make a prior delegation of responsibility for primary presentation and/or primary publication of the results to

the Steering Committee. No other publication is allowed before the primary publication. Any presentation or publication by any trialist must mention the trial and has to be approved by the Steering Committee. Moreover, it is mandatory to make reference to the primary publication.

15 Protocol Amendments

It is specified that the appendices attached to this protocol and referred to in the main text of this protocol, form an integral part of the protocol.

The Investigator should not implement any deviation from, or changes of the Clinical Trial Protocol without agreement by the Sponsor and prior review and documented approval/favorable opinion from the IRB/IEC of an amendment, except where necessary to eliminate an immediate hazard(s) to Clinical Trial Patients, or when the change(s) involves only logistical or administrative aspects of the trial. Any changes agreed upon will be recorded in writing, the written amendment will be signed by the Investigator and by the Sponsor and the signed amendment will be filed with this Clinical Trial Protocol.

Any amendment to the Clinical Trial Protocol requires written approval/favorable opinion by the Ethics Committee (IRB/IEC) prior to its implementation, unless there are overriding safety reasons.

In some instances, an amendment may require a change to the Informed Consent Form. The Investigator must receive an IRB/IEC approval/favorable opinion concerning the revised Informed Consent Form prior to implementation of the change.

16 Bibliographic References

1. Goldman S, Copeland J, Moritz T et al. Starting aspirin therapy after operation. Effects on early graft patency. Department of Veterans Affairs Cooperative Study Group. Circulation. 1991;84(2):520-6.
2. Fremes SE, Levinton C, Naylor CD, Chen E, Christakis GT, Goldman BS. Optimal antithrombotic therapy following aortocoronary bypass: A meta-analysis. Eur J Cardiothroac Surg. 1993;7:169-80.
3. FitzGibbon GM, Kafka HP, Leach AJ, Keon WJ, Hooper GD, Burton JR. Coronary bypass graft fate and patient outcome: angiographic follow-up of 5,065 grafts related to survival and reoperation in 1,388 patients during 25 years. J Am Col Cardiol. 1996;28(3):616-26.
4. Campeau L, Enjalbert M, Lesperance J, Vaislic C, Grondin CM, Bourassa MG. Atherosclerosis and late closure of aortocoronary saphenous vein grafts: sequential angiographic studies at 2 weeks, 1 year, 5 to 7 years and 10 to 12 years after surgey. Circulation. 1983;68(3 Pt 2):II1-II17.
5. Prevent IV Investigators. Efficacy and safety of edifoligide, an E2F transcription factor decoy, for prevention of vein graft failure following coronary artery bypass graft surgery. J Am Med Assoc. 2005;294:2446-54.
6. Singh SK, Desai ND, Chikazawa G, Tsuneyoshi H, Vincent J, Zagorski BM, Pen V, Moussa F, Cohen GN, Christakis GT, Fremes SE. The Graft Imaging to Improve Patency (GRIIP) Clinical Trial Results. J Thorac Cardiovasc Surg (in press).
7. Desai NDD, Cohen EA, Naylor CD, Fremes SE for the Radial Artery Patency Study Investigators. A randomized comparison of radial-artery and saphenous-vein coronary bypass grafts. N Eng J Med. 2004;351:2302-9
8. Hannan EL, Racz MJ, Walford G, et al. Long-Term Outcomes of Coronary-Artery Bypass Grafting versus Stent Implantation. N Engl J Med. 2005;352:2174-83.
9. Hannan EL, Wu C, Walford G, et al. Drug-Eluting Stents vs. Coronary-Artery Bypass Grafting in Multivessel Coronary Disease. N Engl J Med. 2008;358:331-41.
10. Bravata DM, Gienger AL, McDonald KM, et al. Systematic review: the comparative effectiveness of percutaneous coronary interventions and coronary artery bypass graft surgery. Ann Intern Med. 2007;147(10):703-16
11. Daemen J, Boersma E, Flather M, et al. Long-term safety and efficacy of percutaneous coronary intervention with stenting and coronary artery bypass surgery for multivessel coronary artery disease: a meta-analysis with 5-year patient-level data from the ARTS, ERACI-II, MASS-II, and SoS trials. Circulation. 2008;118(11):1146-54.
12. Hlatky MA, Boothroyd DB, Bravata DM, et al. Coronary artery bypass surgery compared with percutaneous coronary interventions for multivessel disease: a collaborative analysis of individual patient data from ten randomised trials. Lancet. 2009;373(9670):1190-7.
13. Serruys PW, Lemos PA, van Hout BA. Sirolimus eluting stent implantation for patients with multivessel disease: rationale for the Arterial Revascularisation Therapies Study part II (ARTS II). Heart. 2004;90(9):995-8.
14. Sousa JE, Costa MA, Sousa AG et al. Two-year angiographic and intravascular ultrasound follow-up after implantation of sirolimus-eluting stents in human coronary arteries. Circulation. 2003;107(3):381-3.

15. Stone GW, Ellis SG, Cox DA et al. One-year clinical results with the slow-release, polymer-based, paclitaxel-eluting TAXUS stent: the TAXUS-IV trial. Circulation. 2004;109(16):1942-47.
16. Naik H, White AJ, Chakravarty T, et al. A meta-analysis of 3,773 patients treated with percutaneous coronary intervention or surgery for unprotected left main coronary artery stenosis. JACC Cardiovasc Interv. 2009;2(8):739-47.
17. Serruys PW, Morice MC, Kappetein AP, *for the SYNTAX Investigators*. Percutaneous Coronary Intervention versus Coronary-Artery Bypass Grafting for Severe Coronary Artery Disease. N Engl J Med. 2009;360:961-72.
18. Desai ND, Naylor CD, Kiss A, et al. Impact of patient and target-vessel characteristics on arterial and venous bypass graft patency. Circulation. 2007;115:684-91.
19. The Post Coronary Artery Bypass Graft Trial Investigators. The effect of aggressive lowering of low-density lipoprotein cholesterol levels and low-dose anticoagulation on obstructive changes in saphenous-vein coronary-artery bypass grafts. N Engl J Med. 1997;336:153-62.
20. Shah SJ, Waters DD, Barter P, Kastelein JJP, Shepherd J, Wenger NK, DeMicco DA. Breazna A, LaRosa JC. Intensive Lipid-Lowering with Atorvastatin for Secondary Prevention in Patients After Coronary Artery Bypass Surgery. J Am Col Cardiol. 2008;51(20):1938-43.
21. Zhao DX, Leacche M, Balaguer JM, et al. Routine intraoperative completion angiography after coronary artery bypass grafting and 1-stop hybrid revascularization results from a fully integrated hybrid catheterization laboratory/operating room. J Am Col Cardiol. 2009;53:232-41.
22. Desai NDD, Miwa S, Kodama D, Cohen G, Christakis GT, Goldman BS, Baerlocher MO, Pelletier MP, Fremes SE. Improving quality of coronary bypass surgery with intraoperative angiography: Validation of a new technique. J Am Col Cardiol. 2005;46:1521-5.
23. Desai ND, Miwa S, Kodama D, Koyama T, Cohen G, Pelletier MP, Cohen EA, Christakis GT, Goldman BS, Fremes SE. A randomized comparison of intraoperative indocyanine green angiography and transit-time flow measurement to detect technical errors in coronary bypass grafts. J Thorac Cardiovasc Surg. 2006;132(3):585-94.
24. VanHimbergen DJ, Koenig SC, Jaber SF, Cerrito PB, Spence PA. A review of transit-time flow measurement for assessing graft patency. Heart Surg Forum. 1999;2(3):226-9.
25. Izzat MB, Khaw KS, Atassi W, Yim AP, Wan S, El Zufari MH. Routine intraoperative angiography improves the early patency of coronary grafts performed on the beating heart. Chest. 1999;115(4):987-90.
26. Loop FD, Lytle BW, Cosgrove DM, Stewart RW, Goormastic M, Williams GW, Golding LA, Gill CC, Taylor PC, Sheldon WC, et al. Influence of the internal-mammary-artery graft on 10-year survival adn other cardiac events. N Engl J Med. 1986;314(1):1-6.
27. Lytle BW, Loop FD, Cosgrove DM, Ratliff NB, Easley K, Taylor PC. Long-term (5 to 12 years) serial studies of internal mammary artery and saphenous vein coronary artery bypass grafts. J Thorac Cardiovasc Surg. 1985;89(2):248-58.
28. Lytle BW, Blackstone EH, Loop FD, Houghtaling PL, Arnold JH, Akhrass R, McCarthy PM, Cosgrove DM. Two internal thoracic artery grafts are better than one. J Thorac Cardiovasc Surg. 1999;117(5):855-72.
29. Taggart DP, D'Amico R, Altman DG. Effect of arterial revascularisation on survival: a systematic review of studies comparing bilateral and single internal mammary arteries. Lancet. 2001;358(9285):870-5.

30. Acar C, Jebara VA, Portoghese M, Beyssen B, Pagny JY, Grare P, Chachques JC, Fabiani JN, Deloche A, Guermonprez JL. Revival of the radial artery for coronary artery bypass grafting. Ann Thorac Surg. 1992;54(2):652-9.
31. Taggart DP, Lees B, Gray A, et al. Protocol for the Arterial Revascularization Trial (ART). A randomized trial to compare survival following bilateral versus single internal mammary grafting in coronary revascularization. Curr Control Trial Cardiovasc Med. 2006;10:118-20.
32. Collins P, Webb CM, Chong CF, Moat NE; Radial Artery Versus Saphenous Vein Patency (RSVP) Trial Investigators. Radial artery versus saphenous vein patency randomized trial: five-year angiographic follow-up. Circulation. 2008;117:2859-64.
33. Hayward PA, Hare DL, Gordon I, Buxton BF. Effect of radial artery or saphenous vein conduit for the second graft on 6-year clinical outcome after coronary artery bypass grafting. Results of a randomised trial. Eur J Cardiothorac Surg. 2008;34:113-7.
34. Damgaard S, Lund JT, Lilleør NB, Perko MJ, Sander K, Dimo B, Jensen MB, Madsen JK, Kelbaek H, Steinbrüchel DA. Comparable three months' outcome of total arterial revascularization versus conventional coronary surgery: Copenhagen Arterial Revascularization Randomized Patency and Outcome trial. J Thorac Cardiovasc Surg. 2008;135:1069-75.
35. Damgaard S, Wetterslev J, Lund JT, et al. One-year results of total arterial revascularization vs. conventional coronary surgery: CARRPO trial. Eur Heart J. 2009:30:1005-11.
36. Muneretto C, Bisleri G, Negri A, et al. Left internal thoracic artery–radial artery composite grafts as the technique of choice for myocardial revascularization in elderly patients: A prospective randomized evaluation. J Thorac Cardiovasc Surg. 2004;127:179-84.
37. Muneretto C, Bisleri G, Negri A, et al. Total arterial myocardial revascularization with composite grafts improves results of coronary surgery in the elderly: a prospective randomized comparison with conventional coronary artery bypass surgery. Circulation. 2003;108(suppl II):II29-II33.
38. Zacharias A, Schwann TA, Riordan CJ Durham SJ, Shah AS, Habib RH. Late results of conventional versus all-arterial revascularization based on internal thoracic and radial artery grafting. Ann Thorac Surg. 2009;87:19-26.
39. Nasso G, Coppola R, Bonifazi R, Piancone F, Bozzetti G, Speziale G. Arterial revascularization in primary coronary artery bypass grafting: Direct comparison of 4 strategies - Results of the Stand-in-Y Mammary Study. J Thorac Cardiovasc Surg. 2009;137:1093-100.
40. Miwa S, Desai N, Koyama T, Chan E, Cohen EA, Fremes SE; Radial Artery Patency Study Investigators. Radial artery angiographic string sign: clinical consequences and the role of pharmacologic therapy. Ann Thorac Surg. 2006;81(1):112-8.
41. Shackcloth MJ, Conant AR, Thekkudan J, Ghotkar S, Simpson AW, Dihmis WC. Attenuation of receptor-dependent and -independent vasoconstriction in the human radial artery. Eur J Cardiothorac Surg. 2008;34:839-44
42. Ding R, Feng W, Li H, Wang L, Li D, Cheng Z, Guo J, Hu D. A comparative study on in vitro and in vivo effects of topical vasodilators in human internal mammary, radial artery and great saphenous vein. Eur J Cardiothorac Surg. 2008;34:536-41.
43. The Parisian Mediastinitis Study Group. Risk factors for deep sternal wound infection after sternotomy: a prospective, multicenter study. J Thorac Cardiovasc Surg. 1996;111(6):1200-7
44. Guru V, Fremes SE, Tu JV. How many arterial grafts are enough? A population-based study of midterm outcomes. J Thorac Cardiovasc Surg. 2006;131:1021-28.

45. Souza DSR, Dashwood MR, Tsui JCS, et al. Improved patency in vein grafts harvested with surrounding tissue: Results of a randomized study using three harvesting techniques. Ann Thorac Surg. 2002;73:1189-95.
46. Souza DSR, Johansson B, Bojo L, et al. Harvesting the saphenous vein with surrounding tissue for CABG provides long-term graft patency comparable to the left internal thoracic artery: Results of a randomized longitudinal trial. J Thorac Cardiovasc Surg. 2006;132:373-8.
47. Dashwood MR, Savage K, Dooley A, et al. Effect of vein graft harvesting on endothelial nitric oxide synthase and nitric oxide production. Ann Thorac Surg. 2005;80:939-44.
48. Dashwood MR, Savage K, Tsui JCS, Dooley A, Shaw SG, Alfonso MSF, Bodin L, Souza DSR.Retaining perivascular tissue of human saphenous vein grafts protects against surgical and distension-induced damage and preserves endothelial nitric oxide synthase and nitric oxide synthase activity. J. Thorac. Cardiovasc. Surg., August 2009; 138: 334 - 340
49. Murphy GJ, Newby AC, Jeremy JY, Baumbach A, Angelini GD. A randomized trial of an external Dacron sheath for the prevention of vein graft disease: The Extent Study. J Thorac Cardiovasc Surg. 2007;134:504-5.
50. Lopes RD, Hafley GE, Allen KB. Endoscopic versus Open-Vein Graft Harvesting in Coronary Bypass Surgery. N Eng J Med. 2009;361:235-44.
51. Bang HO, Dyerberg J, Hjoorne N. The composition of food consumed by Greenland eskimos. Acta Med Scand. 1976;200:69-73.
52. Kris-Etherton PM, Harris WS, Appel LJ and for the Nutrition Committee. Fish Consumption, Fish Oil, Omega-3 Fatty Acids, and Cardiovascular Disease. Circulation. 2002;106:2747-57.
53. Calder PC. n-3 Fatty acids and cardiovascular disease: evidence explained and mechanisms explored. Clin Sci (Lond). 2004;107:1-11.
54. Leon H, Shibata MC, Sivakumaran S, Dorgan M, Chatterley T, Tsuyuki RT. Effect of fish oil on arrhythmias and mortality: systematic review. Brit Med J. 2009;338a:2931-9.
55. Lavie CJ, Milani RV, Mehra MR, Ventura HO. Omega-3 polyunsaturated fatty acids and cardiovascular disease. J Am Col Cardiol. 2009;54:584-94.
56. Thies F, Garry JMC, Yaqoob P, Rerkasem K, Williams J, Shearman CP, et al. Association of n-3 polyunsaturated fatty acids with stability of atherosclerotic plaques: a randomised controlled trial. Lancet. 2003;361:477-85.
57. Din JN, Harding SA, Valerio CJ, et al. Dietary intervention with oil rich fish reduces platelet-monocyte aggregation in man. Atherosclerosis. 2008;197:290–6.
58. Zhao G, Etherton TD, Martin KR, Gilles PJ, West SG, Kris-Etherton PM. Dietary alpha-linolenic acid inhibits proinflammatory cytokine production by peripheral blood mononuclear cells in hypercholesterolemic subjects. Am J Clin Nutr. 2007;85:385–91.
59. O'Keefe JH Jr., Abuissa H, Sastre A, Steinhaus DM, Harris WS. Effects of omega-3 fatty acids on resting heart rate, heart rate recovery after exercise, and heart rate viability in men with healed myocardial infarctions and depressed ejection fractions. Am J Cardiol. 2006;97:1127–30.
60. Geelen A, Brouwer IA, Schouten EG, Maan AC, Katan MB, Zock PI. Effects of n-3 fatty acids from fish on premature ventricular complexes and heart rate in humans. Am J Clin Nutr. 2005;81:416-20
61. Mozaffarian D, Prineas RJ, Stein PK, Siscovick DS. Dietary fish and n-3 fatty acid intake and cardiac electrocardiographic parameters in humans. J Am Col Cardiol. 2006;48:478–84.

62. Gruppo Italiano per lo Studio della Sopravvivenza nell'Infarto miocardico. Dietary supplementation with n-3 polyunsaturated fatty acids and vitamin E after myocardial infarction: results of the GISSI-Prevenzione trial. Lancet. 1999;354:447-455.
63. Marchioli, R., Barzi, F., Bomba, E. et al. Early protection against sudden death by n-3 polyunsaturated fatty acids after myocardial infarction: time-course analysis of the results of the Gruppo Italiano per lo Studio della Sopravvivenza nell'Infarto Miocardico (GISSI)-Prevenzione. Circulation. 2002;105:1897-1903.
64. Yokoyama M, Origasa H, Matsuzaki M. et al. Effects of eicosapentaenoic acid on major coronary events in hypercholesterolaemic patients (JELIS): a randomised open-label, blinded endpoint analysis. Lancet. 2007;369:1090-1098.
65. Burr ML, Fehily AM, Gilbert JF, et al. Effects of changes in fat, fish, and fibre intakes on death and myocardial reinfarction: Diet And Reinfarction Trial (DART). Lancet. 1989;2:757-61.
66. GISSI-HF Investigators. Effect of n-3 polyunsaturated fatty acids in patients with chronic heart failure (the GISSI-HF trial): a randomised, double-blind, placebo-controlled trial. Lancet. 2008;372:1223–30.
67. Rauch B et al. Highly Purified Omega-3 Fatty Acids for Secondary Prevention of Sudden Cardiac Death After Myocardial Infarction – Aims and Methods of the OMEGA-Study. Cardiovasc Drugs Ther. 2006;20:365-375.
68. Calo L, Bianconi L, Colicicchi F, et al. N-3 Fatty acids for the prevention of atrial fibrillation after coronary artery bypass surgery: a randomized, controlled trial. J Am Col Cardiol. 2005;45:1723-8.
69. Eritsland J, Arnesen H, Gronseth K, Fjeld NB, Abdelnoor M. Effect of dietary supplementation with n-3 fatty acids on coronary artery bypass graft patency. Am J Cardiol. 1996;77:31-36.
70. Athanasiou T, Aziz O, Al-Ruzzeh S, et al. Are wound healing disturbances and length of hospital stay reduced with minimally invasive vein harvest? A meta-analysis. Eur J Cardiothorac Surg. 2004;26:1015-26.
71. Singh SK, Desai ND, Petroff SD, et al. The impact of diabetic status on coronary artery bypass graft patency. Circulation. 2008;118:222-225.
