# Supplementary material for: SUPERIOR SVG: no touch saphenous harvesting to improve patency following coronary bypass grafting (a multi-Centre randomized control trial, NCT01047449)
Source: J Cardiothorac Surg. 2019 May 2;14:85. doi: 10.1186/s13019-019-0887-x (PMC6498551; doi:10.1186/s13019-019-0887-x)
Supplement: Supplementary file 2 — Contains additional data regarding trial methodology, definitions, and supplementary results. (DOCX 57 kb) [file 13019_2019_887_MOESM2_ESM.docx]

**Additional file 2: Table of Contents**

1. Interventions page 2

2. Study Outcomes and Definitions page 3

3. Leg Assessment page 5

4. Additional Statistical Analysis Description, Sample Size Estimates and Systematic Review, Meta-Analysis page 6

5. Postoperative Management page 8

6. Patient Follow-up page 9

7. Supplementary Table 1: Baseline Demographics page 10

7. Supplementary Table 2: Individual Reasons for Ineligibility page 12 8. Supplementary Table 3: Operative Characteristics page 13

9. Supplementary Table 4: Size and Quality of Grafts and Targets in the No Touch and Conventional groups page 14

10. Supplementary Table 5: 1-year Patency of All Grafts as assessed by CT Angiography page 15

11. Supplementary Table 6: Adverse surgical outcomes page 16

11. Supplementary Table 7: Additional Leg Outcomes page 17

12. Supplementary Table 8: Randomized clinical trials and observational studies examining No Touch (NT) technique to conventional (CON) saphenous vein harvesting page 19

13. Acknowledgments page 20

14. Appendix II References page 22

**Interventions**

In the no touch (NT) approach, patients had their saphenous vein graft (SVG) harvested using an open atraumatic technique with its surrounding tissue (pedicle). Instead of manual dilation, the harvested vein was left in-situ until required and allowed to dilate and check for bleeding branches only when exposed to arterial pressure, typically when attached to the arterial cannula, or following proximal anastomosis during off-pump surgery. In contrast, patients randomized to the conventional (CON) technique had their SVG harvested in a skeletonized fashion stripping the vein of its adventitial layer. It was then excised and distended using heparinized saline. To reflect contemporary trends in practice, the CON technique could be performed open or endoscopic. The NT or CON study SVG was directed to the most important target for vein grafting as deemed by the surgeon before surgery. Patients underwent duplex scan vein mapping before surgery to assess vein quality and prevent flaps during harvesting. In cases of poor vein quality, the thigh was used to harvest the SVG using the respective techniques. All patients had the left anterior descending artery grafted with an internal mammary artery. Additional arterial grafts were used at the discretion of the operating surgeon. By protocol, the allocated SVG harvesting technique was encouraged to be used as well for additional SVGs. For standardization, all participating Canadian and International centres received NT harvesting training by Dr. Stephen E. Fremes and Dr. Domingos de Souza, respectively.

For the surgical arm, surgeons were unblinded while participants, care providers, data collectors and outcome adjudicators were blinded.

**Study Outcomes and Definitions**

#### Primary outcome

The proportion of study SVGs which are totally (100%) occluded on 64-slice cardiac CT angiography at 1-year post-CABG or death (due to cardiovascular or unknown causes).

#### Secondary outcomes

i. The number of study SVGs with a significant (50-99%) stenosis on 1-year CTA.

ii. The number of study SVGs with a significant stenosis or total occlusion at 1-year CTA.

iii. The incidence of and severity of adverse SV harvesting events by 1-year (infection, haematoma, swelling, neuropathy,).

iv. The incidence of the major adverse cardiac and cerebrovascular events (MACCE, defined as the composite endpoint of all-cause mortality, perioperative and 1-year non-fatal MI (using the WHO definition, see below), stroke, and repeat revascularization (redo CABG or PCI).

**Definitions**

**Cardiovascular (CV) death:** All deaths in the first 30 days are considered CV deaths. All deaths after the first 30 days are considered CV deaths unless a specific non-cardiovascular cause is evident and considered to be the cause of death (e.g. malignancy). All deaths of unknow cause are considered cardiovascular deaths. Furthermore, patients who die during the index hospitalization but after the initial 30 days period (for example long ICU stay with sepsis) will be considered as CV deaths.

**Myocardial infarction:** The pathophysiology of myocardial injury sustained perioperatively (Type V) is likely different from that when injury is sustained later. Early injury can be a manifestation of either graft occlusion, or of a more general insult secondary to ischemia reperfusion, inflammation, and coronary embolization or spasm. Late injury is likely much more similar to the traditional acute coronary syndrome/atherosclerotic process with native vessel or graft occlusion. The definitions for these events differ as below:

(a) Early perioperative myocardial infarction (within 72 hours of surgery):

**WHO Definition^1^:**

1. A CK-MB measurement ≥ 5 times the upper limit of normal with either:
   1. New pathological Q waves or new LBBB (Q wave MI) or
   2. Angiographic evidence of graft occlusion or native coronary artery occlusion or
   3. Imaging evidence of new loss of viable myocardium.

(b) Late perioperative myocardial infarction (later than 72 hours after surgery): ECG changes consistent with myocardial infarction (new significant Q waves in two contiguous leads) or evolving ST-segment or T-wave changes in two contiguous leads signifying ischemia or new left bundle branch block or ST segment elevation and elevated cardiac markers (troponins or CK-MB) in the necrosis range. Myocardial injury occurring after a PCI are included in the late perioperative Myocardial Injury group but are defined as elevation of cardiac markers ≥ 3 times upper limit of normal within 24 hours of PCI or characteristic evolution of new ECG changes.

**Stroke:** Diagnosis of stroke is defined as a focal neurological symptom with rapid onset, lasting at least 24 hours. It is strongly recommended (but not required) that an imaging procedure such as a CT scan or MRI be performed. All strokes will be classified as definite ischemic, definite hemorrhagic or type uncertain. A vascular imaging procedure such as a carotid ultrasound is recommended whenever possible (but not required) for sub-classification of ischemic strokes into cardioembolic, lacunar or large artery.

**Repeat coronary revascularization:** New CABG procedure or PCI associated with documented ischemia by ECG and graft failure or new culprit lesion (≥ 70% luminal stenosis).

**Leg Assessment**

The Leg Assessment Form,^2^ measured at 30-days, 3-months and 1-year, consists of 2 parts: 1 part is functional assessing the quality of life and the other is adverse SVG harvesting events. The assessment is completed for the left leg, the right leg, or both as required. If both legs were used, the worst results were reported.

The quality of life section of the leg assessment consists of 11 questions assessing: Heaviness, Weakness, Stiffness, Itching, Tingling sensation, Heat or burning sensation, Numbness, Pain, Discoloration, Rash or Eczema, Swelling. Each question has a score of 0 (None), 1 (Mild), 2 (Moderate), or 3 (Severe). Responses to each question in the leg assessment quality of life is added for a total quality of life score (range 0-33).

The adverse SVG harvesting events section of the leg assessment includes necrosis, dehiscence, wound drainage, fluid collection, and infection. Necrosis and dehiscence are both assessed with scores 0-3 for none, mild, moderate, or severe. A score is given for both length and width for each so a total score of 0-6 is possible for the necrosis and dehiscence endpoints. Drainage of wound and fluid collection are both assigned a score of 0 if no and 1 if yes.

Infection is assigned a score of 0 if no. If yes, infection is assigned a score of 1-3 for low, moderate or severe as well as a score for treatment of 0-7 for no treatment, topical antibiotics, oral antibiotics, dressings, readmission for leg wound infection, IV antibiotics, debridement, and VAC. Patients that had more than one type of treatment for the infection are coded as the highest code in their combination of treatment. Therefore, the range for infection will be 0-10. The total adverse event score ranges from 0-24 (necrosis 0-6, dehiscence 0-6, drainage 0-1, fluid collection 0-1, infection 0-10).

The total leg score is defined as the sum of the total leg quality of life score and the total adverse event score, range 0-57.

**Additional Statistical Analysis Description, Systematic Review, Meta-Analysis and Sample Size Estimates**

**Statistical Analysis:** Exploratory analyses were performed for the outcomes of any SVG occlusion, or any SVG occlusion or significant stenosis, using generalized estimating equations (GEE) in the two treatment groups to account for correlation in repeated measures in the same patient with an autoregressive correlation matrix^3^. The fixed effects that were included in the model were number of saphenous vein grafts (1, 2 or >=3), treatment group, and the interaction term between group and number of grafts, with the patient specified as a random variable and number of grafts as clusters within subject. Treatment effects are summarized by odds ratios with 95% confidence intervals, provided by the logit as the link function.

A linear mixed model with repeated measures over time (30 days, 3 months and 1 year) was used for the outcome of the total leg score between the treatment groups. The fixed effects were time, treatment, and the interaction term between time and treatment. An autoregressive covariance matrix was used to account for the within-subject correlation. The least squares means for the scores at different times are presented and the pair-wise differences between the treatment groups are reported (Tukey-adjusted p-values)^4^.

**Sample Size:** For the surgical arm, a sample size of 615 patients in each group would provide 80% power for a 2-tailed alpha of 0.05, to identify a relative risk reduction of 30% (NT: 14%, CON: 20%) for study graft occlusion. For the pharmacological arm, a sample size of 540 in each group would provide 80% power to detect a relative risk reduction of 25% (Fish Oils: 22.5%, Placebo: 30%) for patients with >1 graft occlusion. After adjusting for potential protocol violations and loss to follow-up, the corrected sample size was 769 patients in each group (total sample size 1550) to reach adequate statistical power for each arm of the study.

*The sample size was estimated based on graft occlusion. A key limitation with graft occlusion studies is that graft angiography is not performed in patients who died. The primary outcome therefore includes cardiovascular deaths – the sample size calculations were not adjusted further as the numbers of deaths at 1 year is expected to be small, and not different between groups.*

**Systematic Review and Meta-Analysis:** A systematic review of the literature was undertaken and Pubmed was searched from inception to November 2017 using the following key words: "saphenous" AND ("pedicle" OR "no-touch") for studies comparing NT to CON on any of the following outcomes: graft occlusion, graft stenosis, or death. Relevant studies reported SVG graft occlusion 1-2 years postoperatively. The graft occlusion, and graft stenosis or occlusion results of each included study were summarized as odds ratios with the 95% confidence interval. A random effects meta-analysis was performed using the Mantel-Haenszel method. All results were reported with 95% confidence intervals as pooled weighted results of the two subgroups (randomized and observational) and overall. Heterogeneity was assessed using the I^2^ statistic based on the Cochran’s Q statistic, which was further classified as: low (I^2^=0-25%), moderate (I^2^ =26-50%), or high (I^2^>50%). Review Manager (Revman version 5.2; Cochran Collaboration, Oxford, United Kingdom) was used for all analyses.

**Post-operative management**

In this trial, the concomitant medication data was available for everyone at hospital discharge and for 249 (99.6%) (122/123 in the Conventional group) for patients at 1 year follow-up. The overall proportion of patients at discharge and 1-year on aspirin (ASA) was (Discharge: 240 (96.0%), 1-year: 222 (89.2%)), dual antiplatelet therapy (Discharge: 26 (10.4%), 1-year: 20 (8.0%)), statin (Discharge: 227 (90.8%), 1-year: 224 (90.0%)) and β-blockers (Discharge: 224 (89.6%), 1-year (194 (77.9%)). For the surgical arm, medications at discharge and at 1-year were similar between NT and CON; beta-blocker use trended to be lower at discharge in the NT group (NT 109 (85.8%), CON 115 (93.5%), p=0.047) but similar at 1-year (p=0.77).

**Patient Follow-up**

Overall, patients underwent assessment of their grafts at 1-year after surgery using a 64-slice cardiac computed tomography (CCTA). All images were recorded on compact discs and transferred to a central system read by a blinded chest radiologist (LJJ, AZ) or interventional cardiologist with CCTA reading expertise (AA). All patients were also monitored clinically with visits at 30-days, 3, 6, 9, and 12 months post-operatively. Clinical outcomes were all reviewed centrally by an adjudication committee that was blinded to the patients’ treatment allocation.

*Angiographic follow-up*

Of the 250 patients enrolled, 212/250 in the surgical arm (84.8%) (NT: 105, CON: 107) underwent a CCTA at 1-year (Figure 1). The mean time from CABG to CCTA was 12.7 +/- 2.2 months and was similar between NT and CON (p=0.68). Reasons for not undergoing an angiogram are detailed in Figure 1. Of the 212 patients who underwent angiography, 2 patients had no SVG used, and 1 patient had no study SVG (patient withdrew from surgery arm prior to operation) but had SVGs. According to the intention to treat principle, these 3 patients were analyzed for all clinical endpoints in the surgical arm and both angiographic and clinical endpoints in the pharmacological arm.

*Clinical follow-up*

One patient withdrew from the study at 6 months. All other patients had clinical follow-up up to 1 year (mean 13.3 +/- 2.2 months) for major clinical endpoints; follow-up times were similar between groups (NT vs CON, p=0.39).

Table S1: Baseline Demographics

|  | **Surgical Arm** | |
| --- | --- | --- |
|  | **No-Touch**  **(n=127)** | **Conventional**  **(n=123)** |
| **Age (mean +/-SD years)** | 65.5 +/- 9.0 | 64.0 +/- 8.2 |
| **Female n (%)**^≠^ | 21 (16.5) | 10 (8.1) |
| **Body Mass Index (mean +/- SD m/kg^2^)** | 28.2 +/- 4.6 | 29.0 +/- 4.3 |
| **Creatinine (mean +/- SD umol/L)** | 86.5 +/- 19.1 | 89.4 +/- 28.3 |
| **Additive Euroscore (mean +/- SD)** | 3.0 +/- 2.0 | 2.8 +/- 1.8 |
| **Caucasian n (%)** | 95 (74.8) | 93 (75.6) |
| **CCS class** |  |  |
| **1** | 26 (20.4) | 24 (19.5) |
| **2** | 42 (33.1) | 50 (40.7) |
| **3** | 45 (35.4) | 39 (31.7) |
| **4** | 13 (10.2) | 9 (7.3) |
| **NYHA class n (%)** |  |  |
| **1** | 56 (44.1) | 53 (43.1) |
| **2** | 34 (26.8) | 41 (33.3) |
| **3** | 27 (21.3) | 23 (18.7) |
| **4** | 10 (7.9) | 6 (4.9) |
| **Left Ventricular Ejection Fraction**  **(mean +/- SD %)** | 53.2 +/- 11.5 | 52.1 +/- 9.8 |
| **Diabetes n (%)** | 44 (34.6) | 42 (34.1) |
| **Insulin controlled n (%)*** | 20 (45.5) | 12 (28.6) |
| **Hypertension n (%)** | 96 (75.6) | 103 (83.7) |
| **PAD n (%)** | 3 (2.4) | 5 (4.1) |
| **History of smoking** n (%)** | 79 (62.2) | 82 (66.7) |
| **Cerebrovascular Disease n (%)** | 9 (7.1) | 8 (6.5) |
| **Prior Myocardial Infarction n (%)** | 50 (39.4) | 49 (39.8) |
| **Previous Cardiac Surgery n (%)** | 1 (0.8) | 1 (0.8) |
| **Previous PCI n (%)** | 18 (14.2) | 26 (21.1) |
| **Degree of Stenosis (%)** |  |  |
| **Left main > 50%** | 47 (37.0) | 44 (35.8) |
| **LAD > 50%** | 117 (92.1) | 114 (92.7) |
| **Cx > 50%** | 104 (81.9) | 110 (89.4) |
| **RCA > 50%** | 106 (83.5) | 103 (83.7) |

Legend: ≠ P-value – statistically significant between No touch versus Conventional technique - Male (p-value = 0.04). *% of diabetics that are being treated by insulin. **History of former or recent smoking. Abbreviations: CCS - Canadian Cardiovascular Society, NYHA – New York Heart Association, LV grade – left ventricular grade, PAD – peripheral arterial disease, PCI – percutaneous coronary intervention, LAD – left anterior descending artery, Cx – circumflex artery, RCA – right coronary artery, SD – standard deviation

Table S2: Individual Reasons for Ineligibility

| **Total patients not eligible** | **N=1652** |
| --- | --- |
| Emergent CABG | 51 |
| Re-do CABG without all previous grafts occluded | 17 |
| Left ventricular ejection fraction < 20% | 14 |
| SVG not part of revascularization strategy | 214 |
| eGFR < 30 mL/min | 35 |
| Previous vein stripping or poor vein quality | 61 |
| Contraindication to follow-up CT angiography | 33 |
| Pregnant or women of child-bearing age | 3 |
| Allergy to fish oil/fish products or non-medicinal  Ingredients | 4 |
| Already taking fish oil supplements regularly | 41 |
| Congenital or acquired coagulation disorder | 8 |
| Patient considered to be of excessive risk of wound  Infection | 65 |
| Patient not able to provide consent (including language  barrier) | 74 |
| Non-isolated CABG | 761 |
| Patient unavailable to provide consent prior to surgery | 241 |
| More than one reason not eligible | 27 |
| Reason not eligible not specified | 3 |

Table S3: Operative Characteristics

|  |  | | |
| --- | --- | --- | --- |
|  | **No-Touch**  **(n=127)** | **Conventional**  **(n=123)** | **P-value^≠^** |
| **Time from randomization to Surgery (days)- Median (Q1-Q3)** | 0.7 (0.2 -2.8) | 0.7 (0.1-2.0) | 0.61 |
| **Duration of surgery (hours)** | 5.2 +/- 1.6 | 4.8+/- 1.3 | 0.02 |
| **On-pump n(%)** | 123 (96.9) | 120 (97.6) | 0.73 |
| **Cardiopulmonary bypass time (min)** | 105.3 +/- 36.6 | 100.6 +/- 37.3 | 0.33 |
| **Mean cross-clamp time (min)** | 79.4 +/- 34.7 | 75.6 +/- 34.7 | 0.40 |
| **SVG harvested by n(%):** |  |  |  |
| **Assistant Physician** | 35 (27.6) | 40 (32.5) | 0.39 |
| **Physician Assistant** | 30 (23.6) | 26 (21.1) | 0.64 |
| **Senior Resident** | 24 (18.9) | 24 (19.5) | 0.90 |
| **Nurse** | 1 (0.8) | 1 (0.8) | 0.98 |
| **Junior Resident** | 1 (0.8) | 2 (1.6) | 0.54 |
| **Staff Surgeon** | 35 (27.6) | 28 (22.8) | 0.38 |
| **Total grafts per patient – Median (Q1-Q3)** | 3.0 (3.0-4.0) | 3.0 (3.0-4.0) | 0.22 |
| **Type of conduit based on #grafts*** | 403 | 407 |  |
| **SVG n(%)** | 267 (66.3) | 269 (66.1) |  |
| **LIMA n(%)** | 125 (31.0) | 122 (30.0) |  |
| **RIMA n(%)** | 4 (1.0) | 4 (1.0) |  |
| **Radial n(%)** | 7 (1.7) | 12 (2.9) |  |

Legend: *- % is out of total grafts for that group. Abbreviations: SVG – saphenous vein graft, LIMA – left internal mammary artery, RIMA – right internal mammary artery

Table S4: Size and Quality of Grafts and Targets in the No Touch and Conventional groups

|  | **No Touch (N=127)** | **CON (N=123)** |
| --- | --- | --- |
| **Total #Grafts** | 403 | 407 |
| **Size of conduit N (%)*:** |  |  |
| **<1mm** | 2 (0.5) | 1 (0.2) |
| **1-1.5mm** | 20 (5.0) | 17 (4.2) |
| **1.5-2mm** | 164 (40.7) | 160 (39.3) |
| **2-3mm** | 111 (27.5) | 95 (23.3) |
| **3-4mm** | 84 (20.8) | 96 (23.6) |
| **4-5mm** | 21 (5.2) | 28 (6.9) |
| **>5mm** | 1 (0.2) | 10 (2.5) |
| **Quality of conduit N (%)*:** |  |  |
| **Good** | 355 (88.1) | 367 (90.2) |
| **Fair** | 45 (11.2) | 31 (7.6) |
| **Poor** | 3 (0.7) | 9 (2.2) |
| **Target size (%):** |  |  |
| **Small** | 75 (18.6) | 81 (19.9) |
| **Average** | 275 (68.2) | 265 (65.1) |
| **Large** | 53 (13.2) | 61 (15.0) |
| **Target quality N (%)*:** |  |  |
| **Mild plaque** | 229 (56.8) | 246 (60.4) |
| **Moderate plaque** | 106 (26.3) | 111 (27.3) |
| **Severe plaque** | 68 (16.9) | 50 (12.3) |

Legend: The mean number of grafts was similar between No Touch (NT) and Conventional (CON) (NT: 3.2 +/- 0.9, CON: 3.3 +/- 0.9). The size and quality of all of the conduits used were similar between the NT and CON groups as were the quality and size of all of the target vessels. ** % out of total #grafts*

Table S5: 1-year Patency of All Grafts as assessed by CT Angiography

| **Type of graft** | **Total number of grafts** | **Number (%)^a^ of grafts assessed by 1-year CT Angiography** | **Number (%)^b^ of grafts with 100% Occlusion** | **Number (%)^c^ of grafts with 50-99% stenosis or 100% Occlusion** |
| --- | --- | --- | --- | --- |
| **No Touch** | 214 | 191 (89.3) | 18 (9.4) | 19 (9.9) |
| **Open Conventional SVG** | 282 | 243 (86.2) | 36 (14.8) | 46 (18.9) |
| **Endoscopic SVG** | 40 | 30 (75.0) | 3 (10) | 3 (10) |
| **Radial** | 19 | 19 (100.0) | 2 (10.5) | 3 (15.8) |
| **LIMA** | 247 | 204 (82.6) | 4 (2.0) | 6 (2.9) |
| **RIMA** | 8 | 4 (50.0) | 1 (25.0) | 2 (50) |

Legend: Abbreviations: SVG = saphenous vein graft, LIMA = left internal mammary artery, RIMA = right internal mammary artery

a (%) of grafts assessed by 1-yr CT Angiography is out of Total # of grafts.

b (%) of grafts with 100% occlusion is out of # of grafts assessed by 1-yr CT Angiography.

c (%) of grafts with 50-99% stenosis or 100% Occlusion is out of # of grafts assessed by 1-yr CT Angiography

Table S6: Serious Adverse Events

|  | **NT (N=127)**  **N (%)** | **CON (N=123)**  **N (%)** | **P-value** |
| --- | --- | --- | --- |
| Chest reopening | 1 (0.8) | 2 (1.6) | 0.62 |
| Cardiac arrest | 0 | 1 (0.8) | 0.49 |
| IABP | 2 (1.6) | 0 | 0.50 |
| Delirium | 1 (0.8) | 3 (2.4) | 0.36 |
| Transient Ischemic Attack | 0 | 1 (0.8) | 0.49 |
| Sepsis | 0 | 2 (1.6) | 0.24 |
| Pneumonia | 1 (0.8) | 0 | 1.0 |
| Acute Renal Failure | 0 | 1 (0.8) | 0.50 |
| Ischemia Gut | 0 | 1 (0.8) | 0.50 |

Table S7: Additional Leg Outcomes

|  | NT | CON | P-value |
| --- | --- | --- | --- |
| **Incidence of Necrosis (n, %)** |  |  |  |
| 30-days: N = 232 | 11 (9.5%) | 5 (4.3%) |  |
| 3-months: N=206 | 4 (3.8%) | 1 (1.0%) |  |
| 1-year: N= 222 | 0 (0.0%) | 0 (0.0%) |  |
| Cumulative Incidence 1-year | 14 (11.5%) | 6 (5.0%) | 0.070 |
| **Incidence of Dehiscence (n,%)** |  |  |  |
| 30-days: N = 232 | 19 (16.4%) | 11 (9.5%) |  |
| 3-months N=206 | 11 (10.4%) | 2 (2.0%) |  |
| 1-year N= 222 | 0 (0.0%) | 2 (1.8%) |  |
| Cumulative Incidence 1-year: N=241 | 26 (21.3%) | 14 (11.8%) | 0.046 |
| **Wound Drainage (n,%)** |  |  |  |
| 30-days: N = 232 | 17 (14.7%) | 6 (5.2%) |  |
| 3-months N=206 | 11 (10.4%) | 1 (1.0%) |  |
| 1-year N= 222 | 2 (1.8%) | 1 (0.9%) |  |
| Cumulative Incidence 1-year: N=241 | 23 (18.9%) | 7 (5.9%) | 0.002 |
| **Fluid Collection (n,%)** |  |  |  |
| 30-days: N = 232 | 6 (5.2%) | 4 (3.5%) |  |
| 3-months N=206 | 5 (4.7%) | 3 (3.0%) |  |
| 1-year N= 222 | 1 (0.9%) | 0 (0.0%) |  |
| Cumulative Incidence 1-year | 9 (7.3%) | 7 (5.9%) | 0.653 |
| **Incidence of Infection, n (%)** |  |  |  |
| 30-days: N=232 | 27 (23.3) | 11 (9.5) |  |
| 3-months: N= 206 | 10 (9.4) | 3 (3.0) |  |
| 1-year: N=222 | 1 (0.9) | 1 (0.9) |  |
| Cumulative Incidence at 1 year: N= 241 | 31 (25.4%) | 14 (11.8%) | 0.007 |
| **Severity of Infection (Median (Q1-Q3))** |  |  |  |
| 30-daysN= 232 | 0 (0-0) | 0 (0-0) |  |
| 3-months:N=206 | 0 (0-0) | 0 (0-0) |  |
| 1-year:N=222 | 0 (0-0) | 0 (0-0) |  |
| **Adverse SVG Harvesting Event Score (Median (Q1-Q3)** |  |  |  |
| 30-days: N = 232 | 0 (0 – 3) | 0 (0 – 0) |  |
| 3-months N=206 | 0 (0 – 0) | 0 (0 – 0) |  |
| 1-year N= 222 | 0 (0 – 0) | 0 (0 – 0) |  |
| **Leg Quality of Life Score (Median (Q1-Q3)** |  |  |  |
| 30-days: N = 232 | 3 (2 – 6) | 2 (0 – 5) |  |
| 3-months N=206 | 3 (1 – 5) | 2 (0 – 3) |  |
| 1-year N= 222 | 2 (0 – 4) | 1 (0 – 2) |  |
| **Total Leg Score (Median (Q1-Q3))** |  |  |  |
| 30-days N = 232 | 4 (2 – 9) | 3 (1 – 6) |  |
| 3-months N=206 | 3 (1 – 6) | 2 (0 – 3) |  |
| 1-year N= 222 | 2 (0 – 4) | 1 (0 – 2) |  |

Legend: The Adverse SVG Harvesting Event Score is the summation of possible scores of 0-3 for necrosis and dehiscence severity, 0-3 for necrosis and dehiscence length and width, 0-1 for fluid collection and wound drainage, and 0-10 for infection (range 0-24). The Leg Quality of Life assessment consists of 11 questions: Heaviness, Weakness, Stiffness, Itching, Tingling sensation, Heat or burning sensation, Numbness, Pain, Discoloration, Rash or Eczema, Swelling, each rated with a score of 0-3 (range 0-33). The Total Leg Score is the sum of the Adverse SVG Harvesting Event Score and the Leg Quality of Life Scores (range 0-54). The Cumulative Incidence of necrosis, dehiscence, wound drainage and fluid collection at 1 year is defined as any necrosis or dehiscence or wound drainage or fluid collection at 30 days, 3 months or 1year, respectively. Chi-square p-values are provided.

Table S8: Randomized clinical trials and observational studies examining No Touch (NT) technique to conventional (CON) saphenous vein harvesting.

| **Study Name** | **Study Design** | **Primary Outcome** | **Outcomes** | **Odds Ratios (95% CIs)** |
| --- | --- | --- | --- | --- |
| Souza et al. 2002^5^ | Single centre RCT (Sweden) 1993-1997 Patients randomized to Con (n=52) or NT (n=52). | Graft patency (defined as patent or occluded) at mean 18 months follow-up. | **Crude events (occlusions/total # of grafts):**  Con: 12/107  NT: 6/109,  p=0.15 | OR 0.45, (0.15-1.42) |
| Pettersen et al. 2016^6^ | Single centre RCT (Norway) from 2013-2014. Patients randomized to Con (n=50) or NT (n=50). | Intimal hyperplasia as measured by optical coherence tomography at angiography at 6 months follow-up | **Primary outcome:** mean intimal thickness Con 0.36±0.11mm  NT: 0.27±0.06mm, p<0.001  **Crude occlusion rates:**  Con: 6/50  NT: 4/50 | OR: 0.64  (0.17-2.41) |
| SUPERIOR  SVG 2019 (Current Manuscript) | Multi-centre RCT of 12 centres from 2011-2013 randomized patients to Con (n=127) or NT (n=123) in a two by two factorial design where patients were also randomized to fish oil or placebo. | Study graft occlusion or CV death at 1 year. | **Primary outcome:**  Con: 13/123  NT: 7/127,  p=0.14  **Secondary outcomes**  Study graft occlusion:  Con: 11/107  NT: 7/102,  p=0.38  Study graft occlusion or significant stenosis:  Con: 16/107  NT: 8/102,  p=0.11 | OR 0.49 (0.19-1.28)  OR: 0.64, (0.24-1.73)  OR: 0.48, (0.20-1.19) |
| Kim et al. 2016^7^ | Observational study from a single centre (Seoul, Korea) from 2011-2014 using propensity score matching techniques to compare CON (n=98) to NT (n=98) | Early and 1-year angiographic outcomes for graft patency (defined as perfectly patent, patent or occluded). | **Crude events** (occlusions/total grafts) at 1 year.  Con: 17/230  NT: 6/222,  p=0.03 | OR: 0.35 (0.13-0.90) |
| Hu et al. 2017^8^ | Multi-centred randomized trial of NT vs CON (target sample size 2000) | Graft occlusion at 3 months | Recruitment in progress |  |

**ACKNOWLEDGEMENTS**

**Additional Authors:** Michael Raabe MD, Laura Jimenez-Juan MD, Anna Zavodni MD, Tamara Marsden MSc, Jessica Vincent MSc, Roger Baskett MD MSc, Craig D Brown MD, Jean-Francois Morin MD, Erez Kachel MD

**SUPERIOR SVG Committee Members and Investigators**

**INVESTIGATORS:** Investigators who recruited at least 1 patient (number of patients enrolled in each country and site are in parentheses)

CANADA (190) – Hamilton General Hospital (45): R. Whitlock, S. Singh, R. Van Oostveen, A. Kokoszka, T. Creary; University of Alberta Hospital (44): S. Meyer, J. Nagendran, R. MacArthur, J. Mullen, D. Ross, D. Modry, S. Wang, A. Koshal, B. Williams, N. Boroumand, C. Caffrey; Sunnybrook Research Institute (41): S. Fremes, S. Deb, F. Moussa, G. Cohen, R. Karkhanis,; St. Michael’s Hospital (29): S. Verma, D. Latter, L. Errett, M. Peterson, D. Bonneau, K. AlGarni, B. Moran, S. Hill, R. Elituv; St. Boniface General Hospital (12): M. Raabe, R. Arora, M. Moon, A. Munoz, T. Moore, D. Solvason, D. Hrabi; London Health Sciences Centre (11): M. Chu, R. Novick, N. McKenzie, S. Fox, L. Chase; Jewish General Hospital (4): JF. Morin, S. Germain; Maritime Heart Centre (3): R. Baskett, JF. Légaré, G. Hirsh, M. St. Laurent, S. Moore, M. Moeller; New Brunswick Heart Centre (1): C. Brown, M. Pelletier, A. Hassan, R. Forgie, C. Dube, B. Rolfe, S. Brown, T. Campbell, L. Roy; SWEDEN (55) – Orebro University Hospital (43): D. de Souza, M. Drefaldt, M. Arbeus, N. Samano, K. Bergström, K. Gerdevåg; Sahlgrenska University Hospital (12): A. Jeppsson, A. Westerlind, L. Ternström, E. Backlund, C. Roman Emanuel, L. Thimour-Bergström; ISRAEL (5) – Sheba Medical Center (5): E. Kachel, E. Raanani, Y. Kassif, J. Lavee, A. Shinfield, L. Sternik, B. Orlov, H. Cohen, E. Nachum, M. Fakih, F. Daud, D. Spiegelstein, D. Kogan, A. Malachy, E. Gorgatsevich, K. Nachmias.

**POPULATION HEALTH RESEARCH INSTITUTE COORDINATING CENTRE:** K. Brady, J. Vincent, L. Robinson, T. Miranda, P. Rao-Melacini, T. Marsden, S. Pettit, P.J. Devereaux.

**EVENT ADJUDICATION COMMITTEE:**

E.P. Belley-Cote, I. Jaffer, G. Tagarakis, S. Hussain, S. Thelin, E. Duceppe.

**CENTRAL ANGIOGRAPHY ASSESSORS:**

L. Jimenez-Juan, A. Zavodni, A. Al-Saleh

**Appendix II References**

1. Mendis S, Thygesen K, Kuulasmaa K, et al. World Health Organization definition of myocardial infarction: 2008-09 revision. Int J Epidemiol 2011;40:139-46.

2. Verma S, Lovren F, Pan Y, et al. Pedicled no-touch saphenous vein graft harvest limits vascular smooth muscle cell activation: the PATENT saphenous vein graft study. Eur J Cardiothorac Surg 2014;45:717-25.

3. Zeger SL, Liang KY. Longitudinal data analysis for discrete and continuous outcomes.

Biometrics 1986; 42: 121-30

4. McCullagh, P, & Nelder, J. A. (1989). *Generalized Linear Models*, 2nd ed. Chapman & Hall/CRC Press.

5. Souza DS, Dashwood MR, Tsui JC, et al. Improved patency in vein grafts harvested with surrounding tissue: results of a randomized study using three harvesting techniques. Ann Thorac Surg. 2002;73:1189-95

6. Pettersen O, Wiseth R, Hegbom K, Nordhaug DO. Pedicled Vein Grafts in Coronary Surgery Exhibit Reduced Intimal Hyperplasia at 6 Months. J Am Coll Cardiol. 2016;68:427-9.

7. Kim YH, Oh HC, Choi JW, Hwang HY, Kim KB. No-Touch Saphenous Vein Harvesting May Improve Further the Patency of Saphenous Vein Composite Grafts: Early Outcomes and 1-Year Angiographic Results. Ann Thorac Surg. 2017;103:1489-97.

8. Hu S, Wang X. No-Touch Versus Conventional Saphenous Vein Harvesting Technique. Available at ClinicalTrials.gov. <https://clinicaltrials.gov/ct2/show/NCT03126409>. Accessed January 2019.
